# Multimodal Inertial–Visual Sensor Fusion over Evolutionary Deep Temporal Modeling for Humanoid Movement Recognition: A Benchmark Study Toward Sports Telerehabilitation

**DOI:** 10.3390/bioengineering13080866

**Published:** 2026-07-27

**Authors:** Mohammad Shorfuzzaman, Muhammad Hanzla, Bayan Alabdullah, Mohammed Alonazi, Jasem Almotiri, Ahmad Jalal

**Affiliations:** 1 Department of Software Engineering, College of Engineering and Advanced Computing, Alfaisal University, Riyadh 11533, Saudi Arabia; 2 Department of Computer Science, Air University, Islamabad 44000, Pakistan; hanzla0327@gmail.com; 3 Department of Information Systems, College of Computer and Information Sciences, Princess Nourah Bint Abdulrahman University, P.O. Box 84428, Riyadh 11671, Saudi Arabia; bialabdullah@pnu.edu.sa; 4 Department of Information Systems, College of Computer Engineering and Sciences, Prince Sattam bin Abdulaziz University, Al-Kharj 11942, Saudi Arabia; mn.alonazi@psau.edu.sa; 5 Department of Computer Science, College of Computers and Information Technology, Taif University, Taif 21974, Saudi Arabia; j.jasem@tu.edu.sa; 6 Department of Computer Science and Engineering, College of Informatics, Korea University, Seoul 02841, Republic of Korea

**Keywords:** sports telerehabilitation, clinical movement assessment, wearable inertial sensors, multimodal sensor fusion, markerless pose estimation, neuromotor assessment, athlete monitoring, Genetic Algorithm, DeepConvLSTM, humanoid validation benchmark

## Abstract

Wearable inertial sensing and markerless vision are increasingly integrated to enable objective assessment of locomotor and postural function for sports telerehabilitation, intelligent physiotherapy, and athlete performance monitoring. Before such multimodal systems can be translated to clinical practice, their fusion, optimization, and temporal modeling strategies require validation under controlled conditions with reliable ground truth. This study presents a unified multimodal framework that hierarchically integrates inertial measurement unit (IMU) signals and RGB visual information through kernelized representation learning, adaptive multimodal fusion, evolutionary feature optimization, and deep temporal classification. The IMU branch employs Kernelized Extreme Learning Machine (KELM) denoising, Kernelized Canonical Correlation Fusion (KCCF), entropy-guided adaptive windowing, and complementary time-series descriptors (MINIROCKET, TS-CHIEF, and r-STSF). Concurrently, the RGB branch combines anisotropic diffusion filtering, HRNet-based silhouette extraction, DensePose R-CNN, Mesh Graphormer, and Multi-Model Pose-Flow Fusion (MPFF) to learn robust visual representations. Both modalities are integrated through Weighted Canonical Feature Fusion (WCFF) and optimized using a Genetic Algorithm for feature selection and adaptive modality weighting before temporal modeling with cluster-based alignment, Gaussian Process Sequence Modeling, and DeepConvLSTM. As the selected benchmarks do not provide complete inertial recordings, the inertial modality is established according to the adopted experimental protocol to support multimodal fusion analysis. Under 5-fold subject-independent cross-validation, the framework achieves accuracies of 86.56 ± 0.31% on SoccerDiffusion and 88.04 ± 0.25% on HumanoidRobotPose. Although evaluated on humanoid robotic benchmarks, the proposed framework provides a methodological basis for future wearable-enabled clinical movement assessment, remote rehabilitation, and athlete monitoring, while validation on synchronized human inertial-visual datasets remains an important direction for future research.

## 1. Introduction

Artificial intelligence has rapidly transformed the analysis of human movement, enabling objective, quantitative assessment of locomotor and postural function in sports medicine, neuromotor screening, musculoskeletal risk evaluation, and post-operative recovery. Wearable inertial sensing and markerless computer vision have, in particular, shifted movement evaluation out of specialized motion-capture laboratories and toward continuous, in-field, and home-based monitoring. This shift is central to telerehabilitation, in which athletes and patients perform prescribed exercises remotely while sensor-driven systems quantify movement quality, flag compensatory, or injury-prone patterns, and inform clinical decision-making without on-site supervision. By coupling AI-driven sensing with quantitative medical data analysis, such systems can support more reliable clinical decisions and individualized treatment plans, extending the diagnostic and monitoring reach of medical imaging and sensing technologies beyond the clinic and into the patient’s everyday environment. Realizing such systems requires robust recognition and fine-grained characterization of movement, yet this remains exceptionally challenging due to heterogeneous on-body sensing, complex musculoskeletal articulation, and frequent occlusion, illumination change, and viewpoint shifts that degrade single-modality pipelines.

Existing movement-analysis solutions fall into two camps. Sensor-centric approaches exploit wearable IMU and proprioceptive signals efficiently and privately but discard rich appearance and pose cues. Vision-centric approaches leverage convolutional or transformer-based backbones for spatial context; however, they remain vulnerable to occlusion and motion blur and raise privacy concerns in home settings. Multimodal fusion of wearable and visual sensing has become the de facto direction for reliable tele-assessment, but most systems rely on simple concatenation or score-level averaging without discriminative representation optimization, while evolutionary feature selection and cross-modal temporal alignment, both of which are critical when sensor streams are asynchronous and unevenly reliable in unsupervised home environments, remain largely unaddressed.

This paper proposes a hierarchical multimodal framework integrating wearable IMU and RGB streams through kernel-driven fusion, Genetic Algorithm refinement, and a DeepConvLSTM classifier tailored to temporal movement patterns. To permit controlled, reproducible, and privacy-preserving validation prior to human deployment, the framework is evaluated on two humanoid movement benchmarks using the inertial modality described in Section 4.2, achieving accuracies of 86.56% and 88.04% on SoccerDiffusion and HumanoidRobotPose, respectively. The key contributions are as follows:

- A unified multimodal pipeline integrating wearable IMU and RGB streams via KCCF and WCFF, yielding a discriminative cross-modal representation for sensor-driven movement analysis.
- An IMU feature ensemble combining MINIROCKET, TS-CHIEF, and r-STSF with entropy-based windowing to capture diverse temporal movement dynamics.
- An RGB features stream leveraging HRNet, DensePose R-CNN, Mesh Graphormer, and MPFF to jointly model silhouette, structural, and motion-flow information.
- A feature optimization and modeling stage using Genetic Algorithm selection and Gaussian Process sequence learning with cluster-based alignment for robust classification under class imbalance and cross-modal asynchrony.

The remainder of the paper is organized as follows: Section 2 reviews related work in inertial- and vision-based movement analysis. Section 3 presents the proposed methodology with full mathematical formulation. Section 4 reports experiments, ablations, complexity analysis, and comparisons with state-of-the-art methods. Section 5 discusses the findings and their translational implications for telerehabilitation and athlete monitoring, and Section 6 concludes the paper.

## 2. Related Work

Activity recognition for humanoid platforms draws on decades of research into human activity recognition (HAR) and computer vision. To position the proposed framework, this section first reviews IMU sensor-based methods that motivate the left branch of architecture, then surveys RGB-based methods that motivate the right branch and finally discusses why the proposed pipeline is superior to existing systems.

### 2.1. Wearable Sensors for Healthcare

IMU sensors remain the dominant modality for activity recognition due to their privacy-preserving, illumination-invariant, and computationally efficient nature. Demrozi et al. [1] highlighted the transition from hand-crafted features to representation learning and identified limitations of conventional sliding-window methods, motivating the proposed KELM preprocessing and entropy-guided windowing. Chen et al. [2] surveyed deep learning models for sensor-based HAR and characterized their effectiveness for wearable applications, while Ordóñez and Roggen [3] introduced DeepConvLSTM, a widely adopted baseline that serves as the final classifier in this framework. Dempster et al. [4] and MINIROCKET [5], Shifaz et al. [6], and Cabello et al. [7] proposed complementary time-series representations based on convolutional kernels, ensemble trees, and interval-based learning, respectively, which are jointly exploited in the proposed IMU feature ensemble. Complementary wearable-sensor architectures combining convolutional and recurrent models have further improved recognition of complex daily activities [8,9]. Furthermore, Yadav et al. [10] reviewed multimodal HAR and emphasized the benefit of fusing complementary feature representations, while [11] confirmed the effectiveness of proprioceptive signals for motion classification.

### 2.2. RGB Sensors for Healthcare

RGB modalities provide complementary appearance, pose, and contextual information to IMU signals. The cross-view action modeling framework of Wang et al. [12] inspired the parallel processing strategy adopted in the RGB branch, while I3D by Carreira and Zisserman [13] highlighted transferable video representations but motivated lighter pose-aware alternatives for humanoid platforms. HRNet [14] serves as the high-resolution pose estimation backbone, whereas DensePose R-CNN [15] and Mesh Graphormer [16] provide fine-grained articulated and topology-aware representations. Pose Flow [17] inspired the proposed Multi-Model Pose-Flow Fusion (MPFF) for temporally consistent trajectory aggregation. For activity recognition, ST-GCN [18], attentive temporal modeling [19], and TimeSformer [20] demonstrated the effectiveness of spatio-temporal and attention-based representations. In humanoid and human–robot interaction research, the importance of robust multimodal perception, pose-aware temporal sequence alignment, and weakly supervised temporal action segmentation has been emphasized by Liu et al. [21], Zhang et al. [22], and Kuehne et al. [23], respectively, principles that directly underpin the proposed framework.

### 2.3. Recent Advances in Vision-Based and Multimodal Movement Assessment

The requirements of multimodal perception in humanoid robotic platforms differ fundamentally from conventional human activity recognition, as inertial and proprioceptive measurements are acquired from rigid articulated mechanisms with predefined kinematic constraints rather than from soft biological tissues subject to variable sensor placement [24,25]. Recent advances have highlighted the importance of exploiting these structural characteristics through multimodal learning frameworks [26]. In particular, Rozlivek et al. [27] demonstrated that explicitly modeling multimodal information significantly enhances perception in humanoid robots, whereas Haarnoja et al. [28] showed that locomotion and activity classes, including walking, turning, kicking, and fall recovery, can be effectively distinguished using proprioceptive and joint-level observations alone, thereby supporting the activity categories investigated in Section 4.2.1. Complementing these findings, Bin et al. [29] identified viewpoint variation, inter-robot occlusion, and appearance heterogeneity as the principal challenges in visual perception for humanoid robotic systems, while Pavlichenko et al. [30] reported that high-resolution multi-task perception architectures provide robust recognition performance under challenging RoboCup competition environments. Furthermore, the emergence of large-scale self-supervised video representation models, such as VideoMAE V2 [31], has established strong vision-only baselines for video understanding.

### 2.4. Superiority of the Proposed Approach

The proposed framework is built upon several well-established components, including HRNet, DensePose R-CNN, Mesh Graphormer, MINIROCKET, TS-CHIEF, r-STSF, Weighted Canonical Feature Fusion (WCFF), and Genetic Algorithm (GA)-based optimization, none of which is individually claimed as novel. Accordingly, the contribution of this work does not lie in integrating existing algorithms, but in formulating a unified multimodal optimization framework that enables these established components to operate cooperatively under a common learning strategy.

The primary contribution is the joint optimization of adaptive modality balancing and feature selection within a single optimization process. Unlike conventional multimodal approaches that treat feature fusion and feature selection as independent sequential stages, the proposed framework optimizes both simultaneously. Since the discriminative importance of individual features depends on the relative contribution of the inertial and visual modalities, jointly optimizing these variables enables the framework to learn a more compact and discriminative multimodal representation than conventional sequential optimization strategies.

Furthermore, the proposed fusion strategy differs fundamentally from naïve feature concatenation. Rather than simply combining heterogeneous feature descriptors, it adaptively balances modality contributions while emphasizing complementary cross-modal information and suppressing redundant or modality-specific features. The subsequent optimization further refines the fused representation by removing redundant dimensions, allowing multimodal fusion and feature optimization to operate as a unified learning process.

Finally, the proposed multikernel learning strategy adaptively combines complementary kernel functions according to their discriminative capability instead of employing fixed or uniformly weighted kernels. Collectively, these methodological formulations constitute the principal contribution of the proposed framework, whereas the remaining architectural components serve as established building blocks within the overall multimodal learning pipeline.

## 3. Methodology

**Figure 1:**
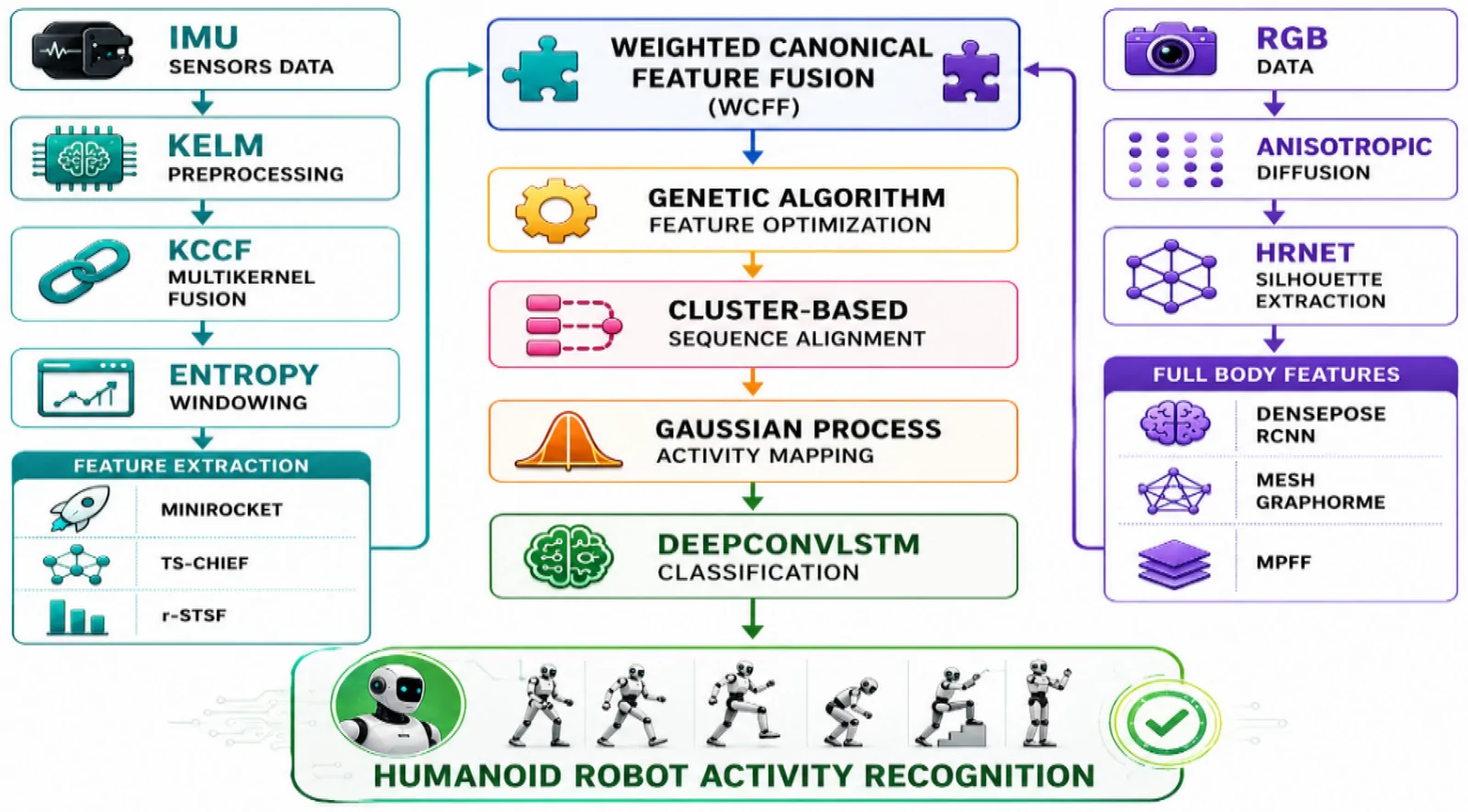
End-to-end pipeline of the proposed multimodal humanoid robotic activity recognition framework. Arrows represent the workflow between successive processing stages, and the different colors are used solely to differentiate the modules visually.

The proposed framework recognizes humanoid robotic activities using two complementary modalities. Each stream is independently processed to extract modality-specific features, which are fused through Weighted Canonical Feature Fusion to learn a shared representation. The fused features are optimized using a Genetic Algorithm, temporally aligned, modeled through a Gaussian Process Sequence Model, and classified by a DeepConvLSTM. Figure 1 illustrates the dual-branch architecture and unified fusion–classification pipeline.

### 3.1. IMU Sensor Processing Branch

#### 3.1.1. Preprocessing via Kernelized Extreme Learning Machine (KELM)

Raw IMU signals are often affected by noise, drift, and sensor interference. To enhance signal quality while preserving motion dynamics [32], a Kernelized Extreme Learning Machine (KELM) is employed for nonlinear denoising. Let $X=\{x_{t}{\}}_{t=1}^{T}\in R^{T\times d}$ represent an IMU sequence, and the KELM output for input $x_{t}$ is computed as:

(1) $$f\left(x_{t}\right)=k\left(x_{t},X\right){\left(K+\frac{1}{C}I\right)}^{-1}Y$$

where $K\in R^{T\times T}$ denotes the kernel matrix with elements $K_{ij}=\kappa \left(x_{i},x_{j}\right)$, Y represents the target output matrix, C is the regularization parameter, and I is the identity matrix. The Gaussian RBF kernel is adopted as:

(2) $$\kappa \left(x_{i},x_{j}\right)=exp\left(-\frac{∥x_{i}-x_{j}{∥}^{2}}{2\sigma^{2}}\right)$$

The Gaussian kernel effectively models highly non-stationary humanoid motions while ensuring a strictly positive-definite kernel matrix. The bandwidth parameter σ is determined through grid search to maximize reconstruction fidelity. The output weights β are obtained in closed form by minimizing the regularized residual sum of squares:

(3) $$\beta^{*}={\left(K+\frac{1}{C}I\right)}^{-1}Y,\;\underset{\beta }{min}\left(∥Y-K\beta {∥}^{2}+\frac{1}{C}∥\beta {∥}^{2}\right)$$

**Figure 2:**
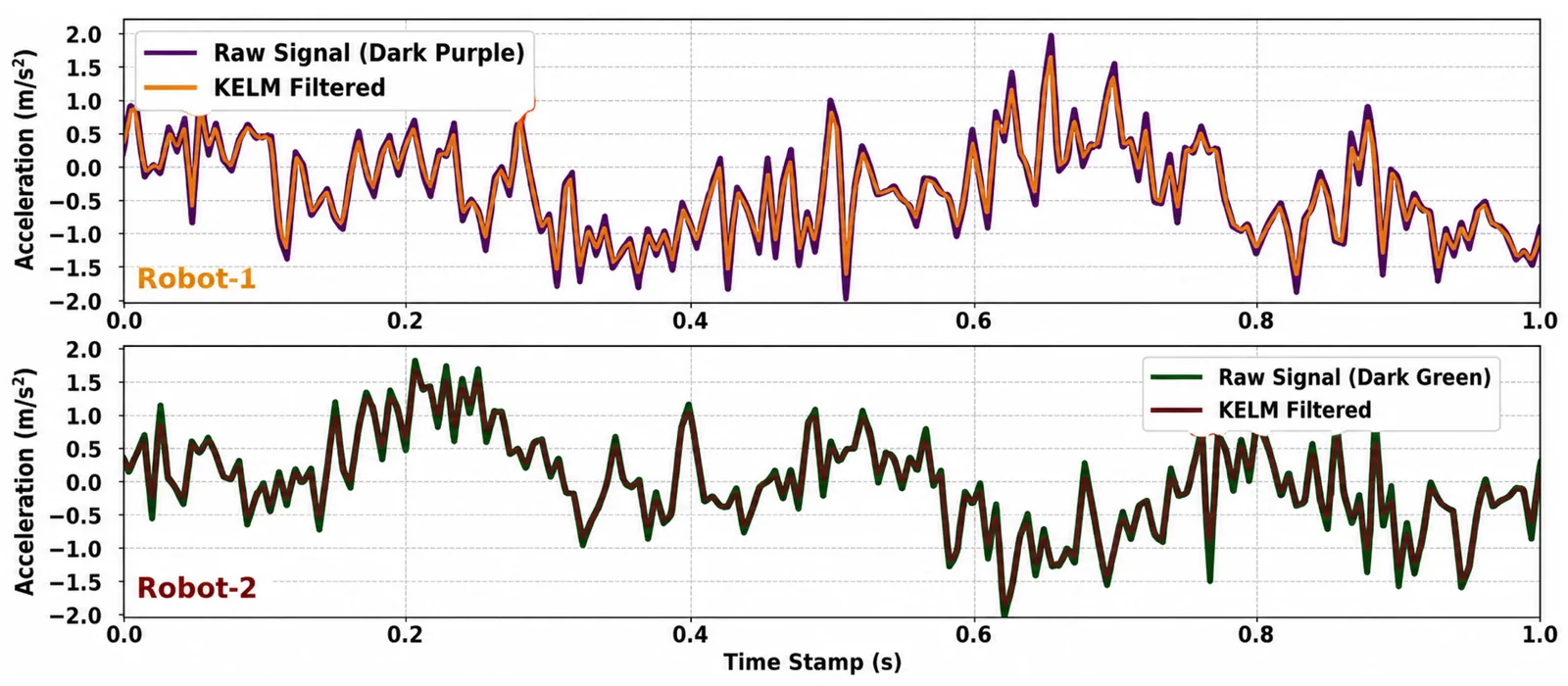
Effect of KELM-based preprocessing on a representative humanoid IMU acceleration channel: raw vs. denoised waveform.

This formulation yields a denoised reconstruction ${\tilde{x}}_{t}=f\left(x_{t}\right)$, preserving discriminative motion characteristics while attenuating high-frequency noise, as illustrated in Figure 2.

#### 3.1.2. Multikernel Fusion via Kernelized Canonical Correlation Fusion (KCCF)

Humanoid robots use multiple IMU sensors to capture complementary motion information. To fuse these heterogeneous signals, Kernelized Canonical Correlation Fusion (KCCF) projects sensor streams into a shared latent space that maximizes nonlinear correlations and models complex kinematic relationships. Given kernel matrices $K_{a}$ and $K_{b}$ for sensor streams $X_{a}$ and $X_{b}$, KCCF learns projection vectors α and β by maximizing kernelized correlation:

(4) $$\rho \left(\alpha ,\beta \right)=\frac{\alpha^{T}K_{a}K_{b}\beta }{\sqrt{\left(\alpha^{T}K_{a}^{2}\alpha \right)\left(\beta^{T}K_{b}^{2}\beta \right)}}$$

For the multi-IMU setting, the formulation is extended to M streams through the following regularized multikernel objective:

(5) $$\underset{\left\{\alpha_{m}\right\}}{max}\sum_{i<j}\alpha_{i}^{T}K_{i}K_{j}\alpha_{j}\;s.t.\;\alpha_{m}^{T}\left(K_{m}^{2}+\lambda I\right)\alpha_{m}=1,\;\forall m$$

The optimization reduces to the generalized eigenvalue problem:

(6) $$\left[\begin{array}{cc} 0 & K_{a}K_{b}\\ K_{b}K_{a} & 0 \end{array}\right]\left[\begin{array}{c} \alpha \\ \beta \end{array}\right]=\Lambda \left[\begin{array}{cc} K_{a}^{2}+\lambda I & 0\\ 0 & K_{b}^{2}+\lambda I \end{array}\right]\left[\begin{array}{c} \alpha \\ \beta \end{array}\right]$$

where Λ denotes the diagonal matrix of canonical correlations. To further enhance discriminative capability, a multikernel composition is adopted by combining Gaussian, Laplacian, and polynomial kernels through non-negative weights $w_{q}$, optimized via gradient descent using a class-separability objective:

(7) $$K_{m}=\sum_{q=1}^{Q}w_{q}K_{m}^{\left(q\right)},\;\;w_{q}\geq 0,\;\;\sum_{q}w_{q}=1$$

**Figure 3:**
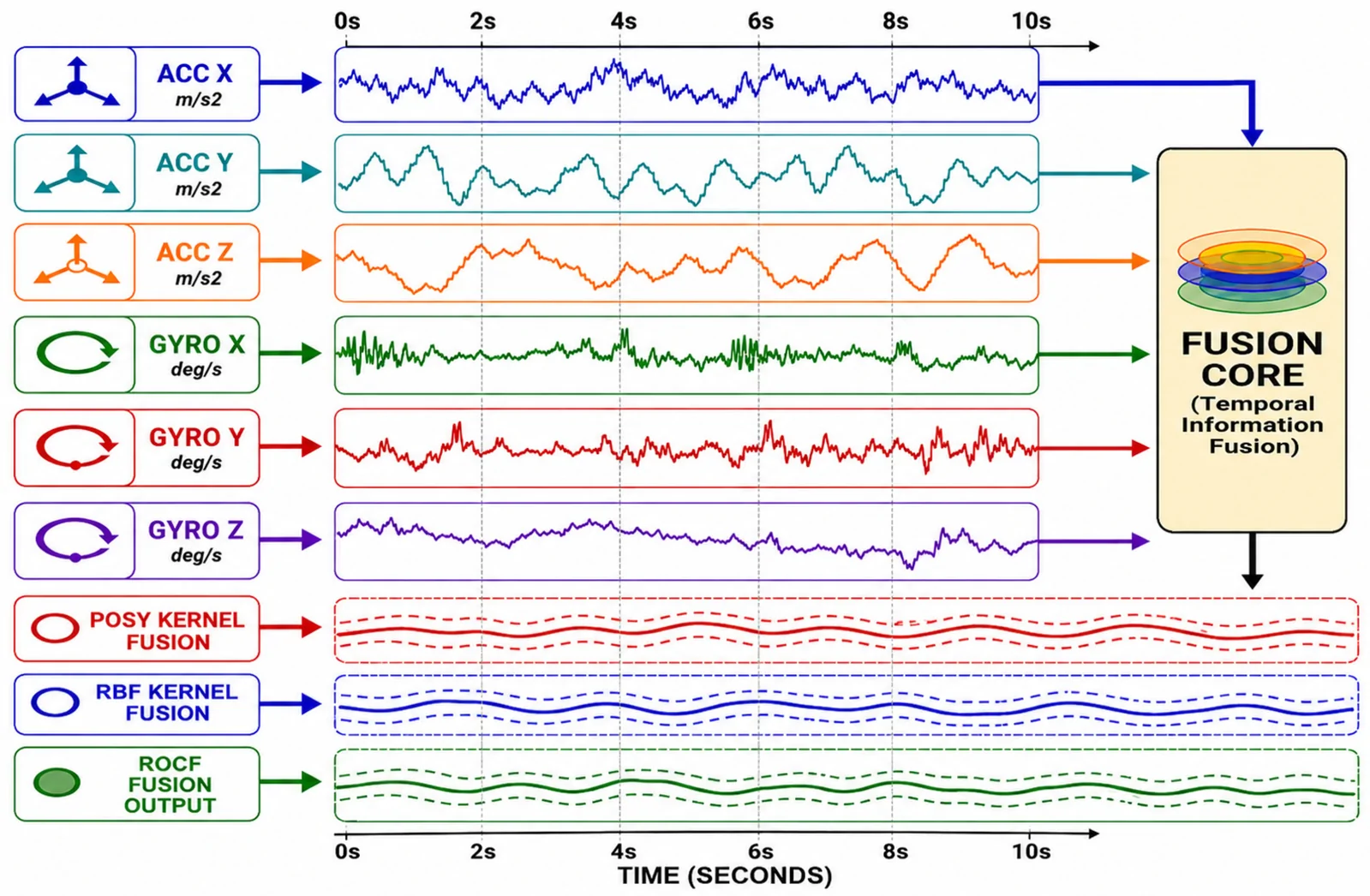
Kernelized Canonical Correlation Fusion (KCCF) of tri-axial accelerometer and gyroscope signals using RBF and Polynomial kernels. Arrows indicate the data flow, solid and dashed lines represent fused signals and their confidence bounds, respectively, while colors distinguish sensor channels and fusion stages.

The optimization is driven by the Fisher discriminant ratio:

(8) $$J_{F}\left(K_{m}\right)=\frac{trace\left(S_{b}\left(K_{m}\right)\right)}{trace\left(S_{w}\left(K_{m}\right)+\varepsilon I\right)}$$

where $S_{b}$ and $S_{w}$ denote the between-class and within-class scatter matrices, respectively, while ε ensures numerical stability. Maximizing $J_{F}$ learns optimal kernel weights, producing a unified discriminative IMU feature representation $Z_{\mathrm{IMU}}$. The output of the KCCF can be depicted in Figure 3.

#### 3.1.3. Windowing via Entropy-Monitoring Windowing

Following KCCF fusion, the IMU representation is segmented into temporal windows for feature extraction. To accommodate variable-duration humanoid actions, an entropy-based adaptive windowing approach is employed instead of fixed-length windows. It dynamically adjusts window size according to signal complexity, producing longer windows for stable motions and shorter windows for transitions. The Shannon entropy of a window $W=\{x_{t}{\}}_{t=t_{0}}^{t_{0}+L}$ is computed as:

(9) $$H\left(W\right)=-\sum_{k=1}^{B}p_{k}\left(W\right)logp_{k}\left(W\right)$$

where $p_{k}$ is the empirical probability of the k-th amplitude bin and B is the total number of bins. A new window boundary is created when the entropy variation exceeds an adaptive threshold:

(10) $$\left|H\left(W_{t}\right)-H\left(W_{t-\Delta }\right)\right|>\tau \sigma_{H},\;\;\tau =0.18$$

**Figure 4:**
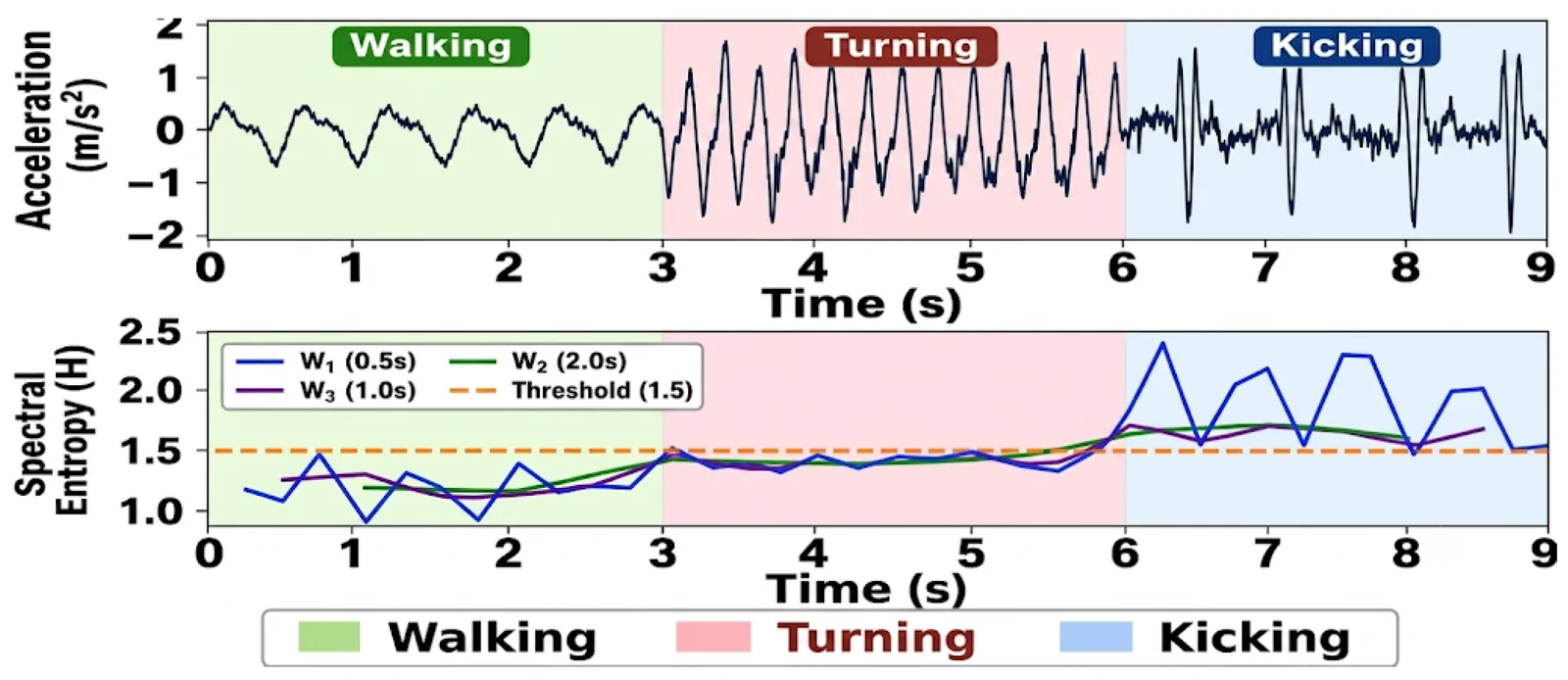
Multi-windowing segmentation via entropy monitoring, illustrating signal transitions between walking, running, and kicking based on spectral entropy thresholds.

This strategy generates information-balanced windows that preserve transient activity boundaries essential for accurate humanoid activity recognition. The windowing results can be seen in Figure 4.

#### 3.1.4. IMU Feature Extraction Methods

Each entropy-based window is processed using three complementary feature extractors: MINIROCKET for convolutional temporal patterns, TS-CHIEF for ensemble-based decision features, and r-STSF for interval-level statistics. This multi-descriptor setup captures both short- and long-term motion dynamics, improving robustness across diverse humanoid activities.

A. MINIROCKET descriptor

MINIROCKET is used to extract local temporal and motion-frequency features from IMU sequences. It has been chosen for its efficient, fixed-kernel design that avoids training while producing discriminative representations. A set of deterministic kernels is applied to each window, and the proportion of positive responses forms the feature representation:

(11) $$\phi_{n}\left(W\right)=\frac{1}{T\prime }\sum_{t=1}^{T\prime }1\left\{{\left(W\times h_{n}\right)}_{t}>b_{n}\right\}$$

where T′=T−8 and $b_{n}$ denotes a kernel-specific bias. To capture motion patterns occurring at different temporal scales, the kernels are evaluated using multiple dilation factors $d\in \{1,2,4,\ldots ,2^{k}\}$. The resulting convolution operation is defined as:

(12) $${\left(W\times h_{n}\right)}_{t}=\sum_{j=0}^{8}h_{n}\left[j\right]\cdot W\left[t+d\cdot j\right]$$

**Figure 5:**
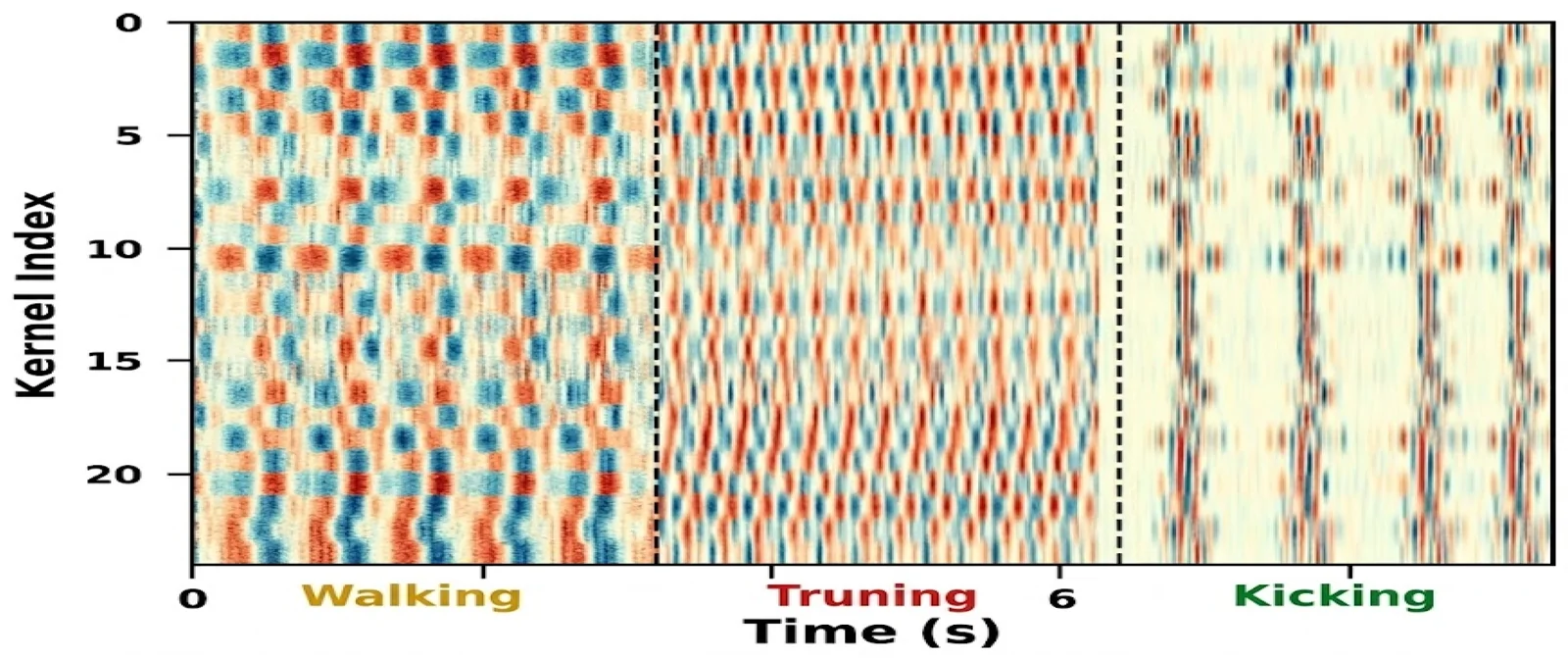
Heatmap of kernel-indexed features over time.

The multi-dilation strategy enables robustness to variations in execution speed and motion frequency, which are common in humanoid activities involving locomotion, manipulation, and posture transitions. The result for the MINIROCKET can be seen in Figure 5.

B. TS-CHIEF descriptor

**Figure 6:**
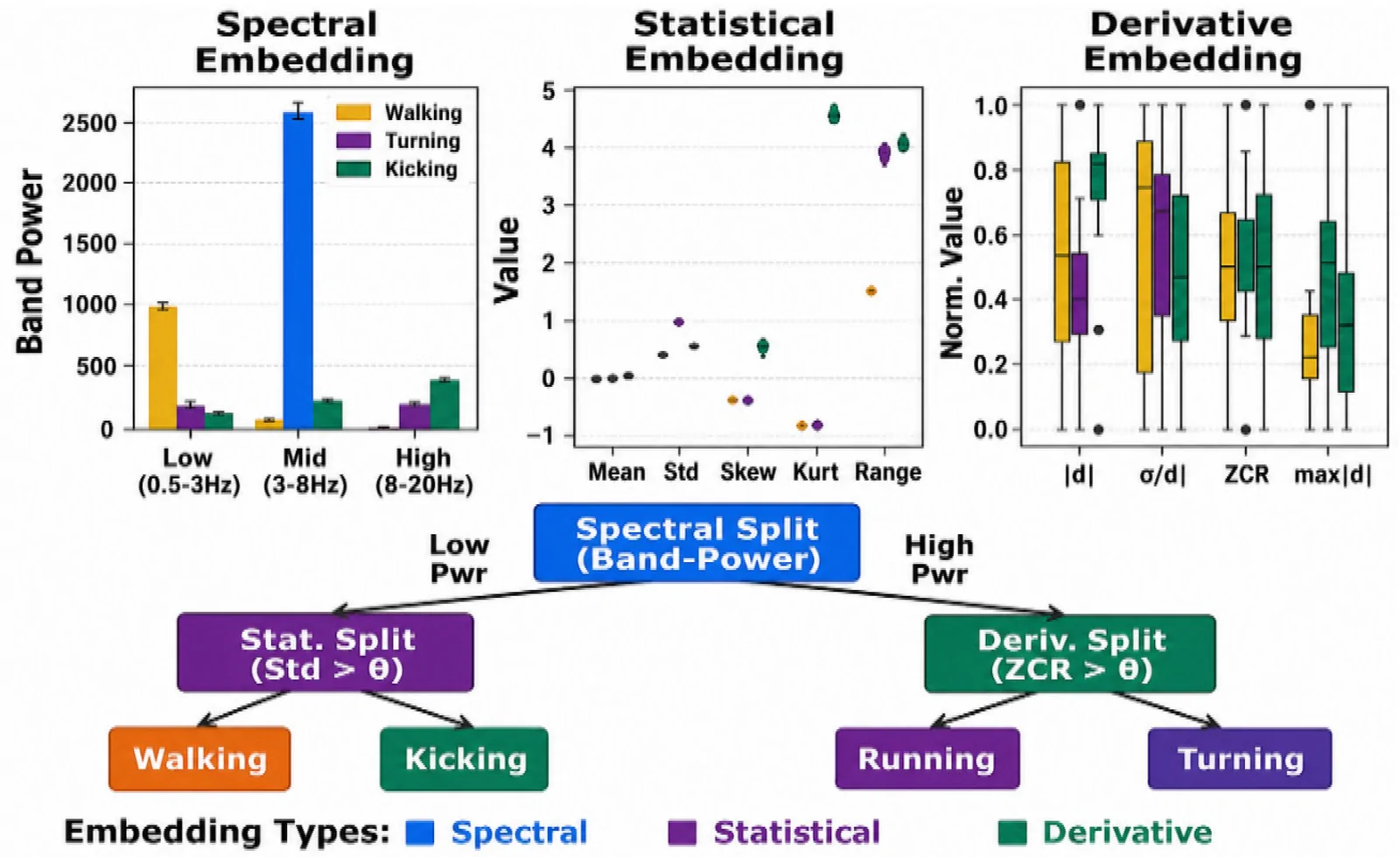
TS–CHIEF heterogeneous embedding forest feature extraction across spectral, statistical, and derivative domains with its corresponding hierarchical decision structure for activity classification. Colors indicate the different embedding types and their associated activity classes.

TS-CHIEF is used to model complex temporal patterns not captured by convolutional features. It integrates multiple time-series representations in an ensemble framework to extract both local and global activity characteristics, which is useful for distinguishing subtle variations in humanoid motion. It builds an ensemble of decision trees using similarity-, dictionary-, and interval-based splits, selecting each node split by maximizing information gain:

(13) $$IG\left(n\right)=H\left(D_{n}\right)-\sum_{c\in \{L,R\}}\frac{\left|D_{n,c}\right|}{\left|D_{n}\right|}H\left(D_{n,c}\right)$$

where $H\left(\cdot \right)$ denotes entropy and $D_{n}$ represents the training samples arriving at node n. Each tree produces a class prediction, while the structural information learned by the ensemble is represented through a leaf-index embedding:

(14) $$T_{emb}\left(W\right)=\left[\delta \left(l_{1}\left(W\right)\right),\delta \left(l_{2}\left(W\right)\right),\ldots ,\delta \left(l_{E}\left(W\right)\right)\right]$$

where $l_{e}\left(W\right)$ denotes the leaf node reached by tree e and $\delta \left(\cdot \right)$ represents one-hot encoding. This embedding preserves the hierarchical decision pathways learned by the ensemble and provides an additional discriminative representation of activity structure. The outcome of the TS–CHIEF can be seen in Figure 6.

C. r–STSF descriptor

The Randomized Supervised Time-Series Forest (r–STSF) captures interval-based statistical patterns in IMU signals, as humanoid actions often differ in their temporal motion statistics. It computes statistical features over randomly sampled intervals, forming the descriptor:

(15) $$\Psi \left(W\right)={\left[\mu \left(W_{a_{k}:b_{k}}\right),\sigma^{2}\left(W_{a_{k}:b_{k}}\right),s\left(W_{a_{k}:b_{k}}\right),\rho \left(W_{a_{k}:b_{k}}\right)\right]}_{k=1}^{K}$$

where μ, $\sigma^{2}$, s, and ρ denote the mean, variance, skewness, and lag–1 autocorrelation over interval $\left[a_{k},b_{k}\right]$, respectively. The discriminative importance of each interval is measured using the Fisher score:

(16) $$F\left(k\right)=\frac{\sum_{c}n_{c}{\left(\mu_{c,k}-\mu_{k}\right)}^{2}}{\sum_{c}n_{c}\sigma_{c,k}^{2}}$$

Higher scores indicate stronger class separability, allowing the selection of the most informative intervals while reducing redundancy. Finally, the features extracted by MINIROCKET, TS–CHIEF, and r–STSF are fused as:

(17) $$F_{IMU}=\left[\phi ;\;T_{emb};\;\Psi \right]$$

**Figure 7:**
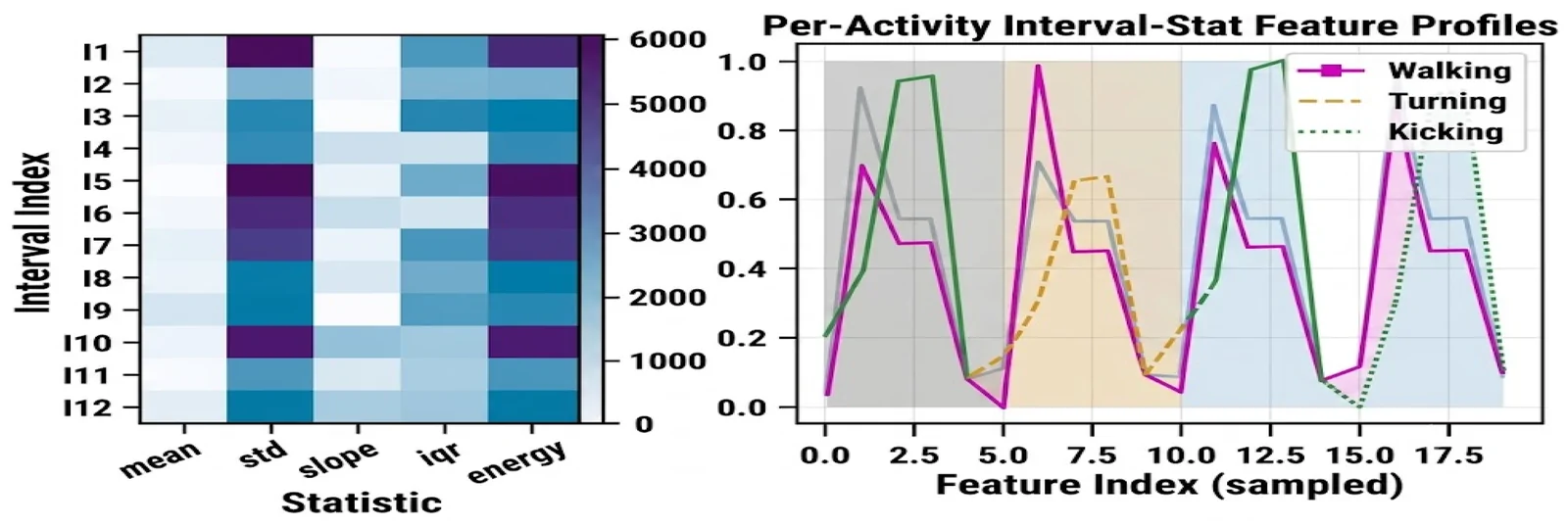
Heatmap of statistical F-values and corresponding per-activity interval feature profiles for walking, turning, and kicking. Colours and line styles distinguish the activity profiles, while the green solid line denotes the overall top-ranked interval profile.

This representation integrates frequency-aware, hierarchical temporal, and interval-statistical information, providing a compact and discriminative description of humanoid motion. The result for the r–STSF is shown on Figure 7.

### 3.2. RGB Visual Processing Branch

#### 3.2.1. Preprocessing via Anisotropic Diffusion

**Figure 8:**
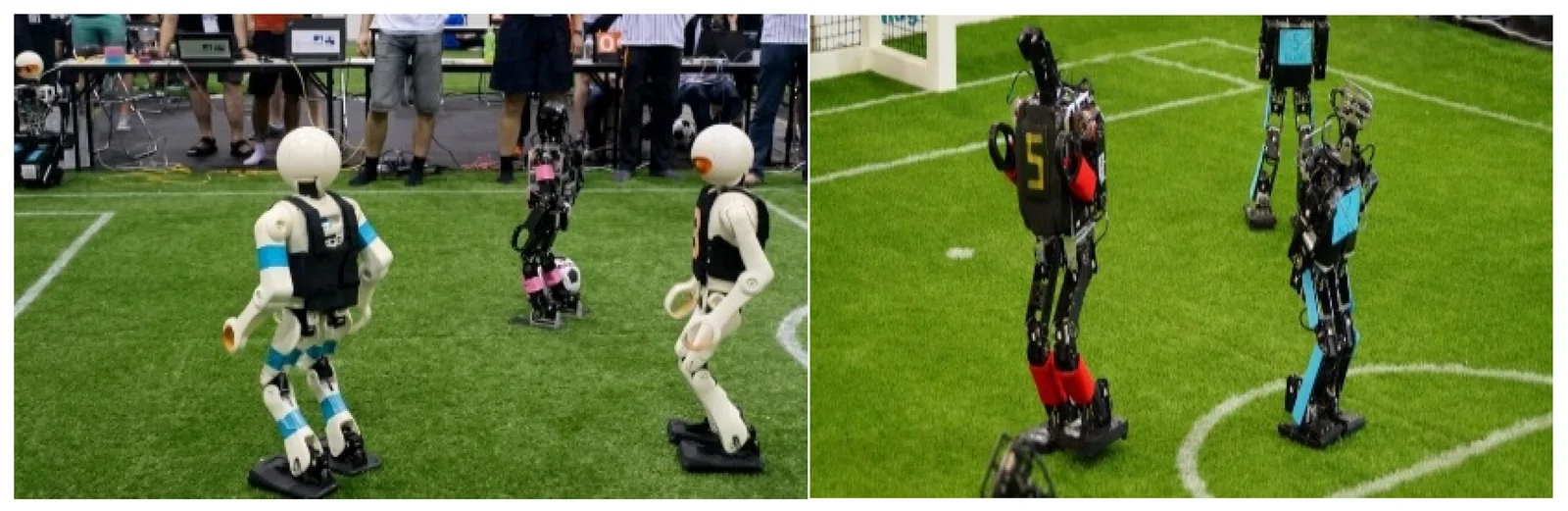
Anisotropic diffusion preprocessing on an edge-preserving denoised output of an RGB frame.

RGB streams from humanoid robots often contain noise, motion blur, and illumination variations that affect pose and silhouette estimation. To address this, anisotropic diffusion (Perona–Malik filtering) is employed to suppress noise while preserving body contours and joint boundaries. The image I(x,y,t) evolves according to the following:

(18) $$\frac{\partial I}{\partial t}=\nabla \cdot \left(c\left(∥\nabla I∥\right)\nabla I\right)$$

where $c\left(\cdot \right)$ denotes the diffusion coefficient, defined as:

(19) $$c\left(∥\nabla I∥\right)=exp\left(-{\left(\frac{∥\nabla I∥}{\kappa }\right)}^{2}\right)$$

with κ controlling edge preservation. The discrete implementation is shown as follows:

(20) $$I_{x,y}^{\left(n+1\right)}=I_{x,y}^{\left(n\right)}+\Delta t\sum_{d\in N_{4}}c\left(\nabla_{d}I_{x,y}^{\left(n\right)}\right)\nabla_{d}I_{x,y}^{\left(n\right)}$$

where $N_{4}$ denotes the four-neighborhood system and $\nabla_{d}$ the directional finite-difference operator. Anisotropic diffusion is applied for 15 iterations with Δt=0.20, producing edge-preserving denoised frames that improve silhouette and pose extraction. The result is shown in Figure 8.

#### 3.2.2. Silhouette Extraction via HRNet

Silhouette information provides a compact representation of body posture for distinguishing similar humanoid activities. To preserve fine spatial details often lost in conventional encoder–decoder architectures, HRNet maintains high-resolution features through multi-scale fusion, improving robustness to occlusion, pose variation, and viewpoint changes [14]. Let $X^{\left(s\right)}$ denote features at scale s and $W^{\left(s\to s^{\prime }\right)}$ denotes the resampling operator. Multi-scale fusion is defined as:

(21) $$X_{k}^{\left(s\prime \right)}=\sum_{s=1}^{S}W^{\left(s\right)\to \left(s\prime \right)}X_{k-1}^{\left(s\right)}$$

The silhouette mask M is generated using a 1×1 convolution and sigmoid activation:

(22) $$M=\sigma \left(Conv_{1\times 1}\left(Concat\left(X_{K}^{\left(1\right)},\ldots ,X_{K}^{\left(S\right)}\right)\right)\right)$$

Training employs a combined binary cross-entropy and Dice loss:

(23) $$L_{seg}=-\sum_{p}\left[y_{p}logM_{p}+\left(1-y_{p}\right)log\left(1-M_{p}\right)\right]+\alpha \left(1-\frac{2\sum_{p}y_{p}M_{p}}{\sum_{p}y_{p}+\sum_{p}M_{p}}\right)$$

**Figure 9:**
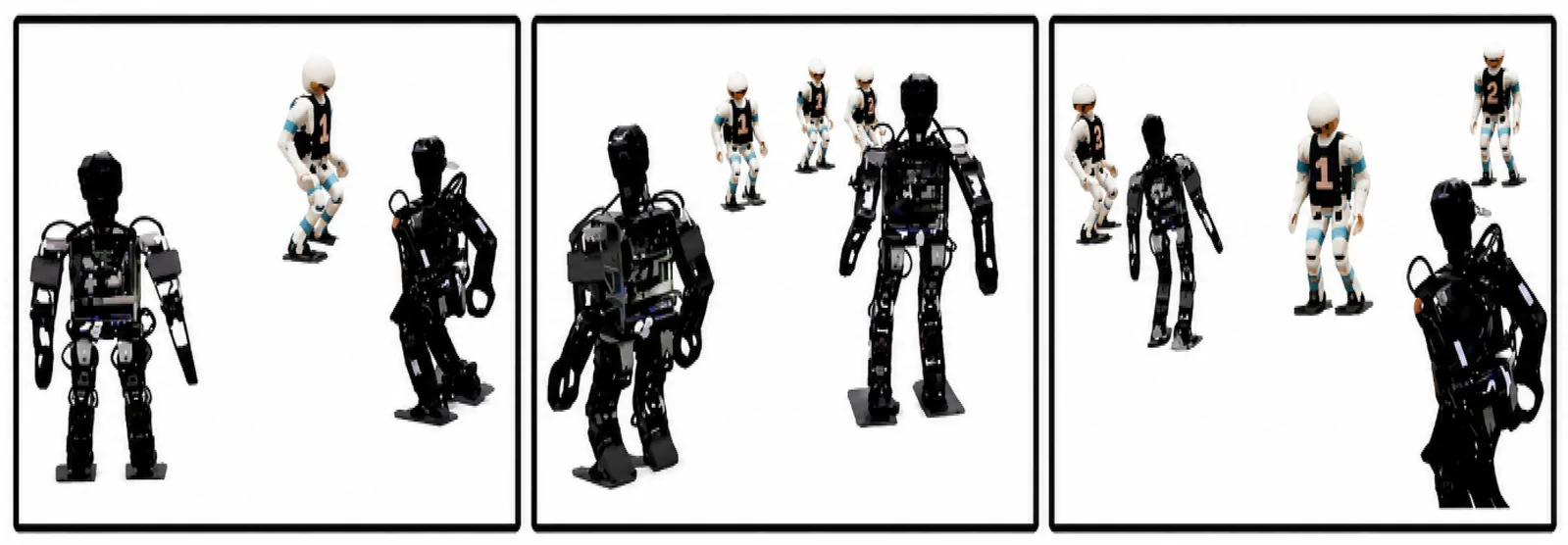
Visual setup for multi-robot silhouette extraction and segmentation using HRNet.

This loss enhances pixel accuracy and mask completeness. HRNet-W32, pretrained on COCO and fine-tuned on humanoid silhouettes, produces high-resolution masks for subsequent feature extraction. The output of the HRNet can be seen in Figure 9. 

#### 3.2.3. RGB Feature Extraction Methods

After silhouette extraction, three visual descriptors are used: DensePose R-CNN for body surface mapping, Mesh Graphormer for structural pose relations, and MPFF for temporal motion. These complementary features capture geometry, structure, and dynamics, which single methods often miss.

A. DensePose R-CNN

**Figure 10:**
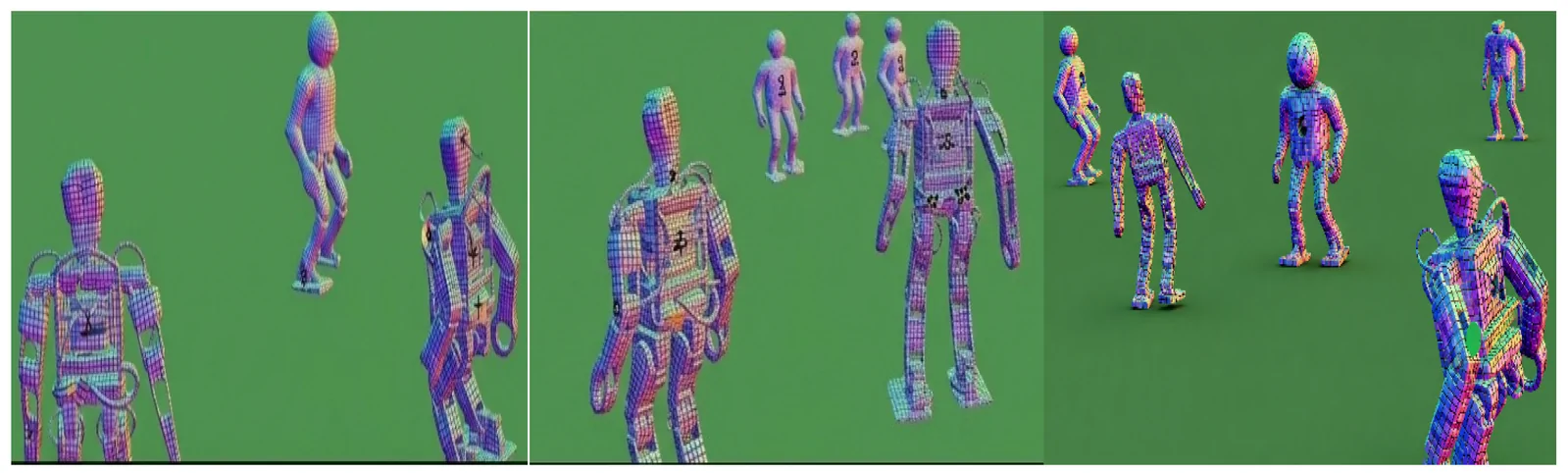
DensePose R-CNN surface mapping. Dense 3D mesh estimation and UV coordinate mapping on the humanoid robot subjects.

DensePose R-CNN extracts dense pixel-to-surface correspondences for detailed body-part localization, providing richer spatial information than sparse keypoint methods. This enables accurate modeling of subtle limb articulations and fine-grained humanoid movements. The DensePose regression objective is defined as:

(24) $$L_{DP}=\sum_{p\in S}∥\left(u_{p},v_{p}\right)-\left({\hat{u}}_{p},{\hat{v}}_{p}\right){∥}^{2}+\mu_{cls}L_{part}\left(p\right)$$

where $L_{part}\left(p\right)$ denotes the body-part classification loss and $\mu_{cls}$ balances the regression and classification objectives. The aggregated DensePose feature representation is computed through ROI pooling:

(25) $$F_{DP}=ROIPool\left(\Psi_{DP}\left(I_{seg}\right),R_{t}\right)$$

where $R_{t}$ denotes the detected regions of interest at frame t. The resulting descriptor captures detailed surface-level posture information that complements higher-level skeletal representations. The result of the DensePose R-CNN can be seen in Figure 10.

B. Mesh Graphormer

**Figure 11:**
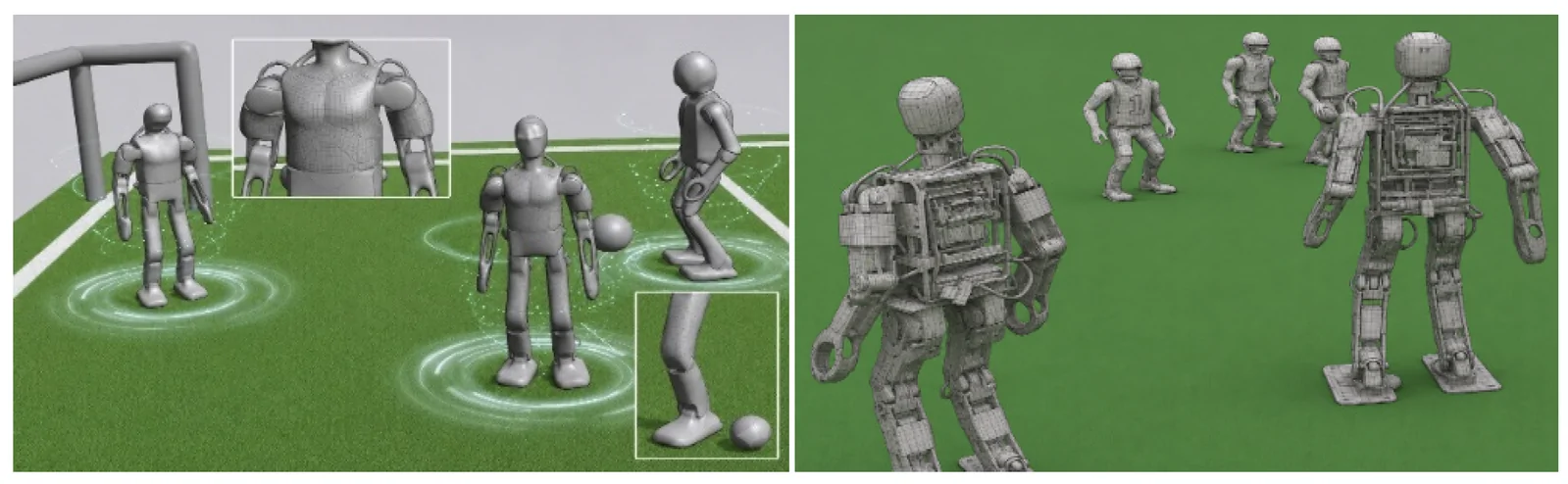
3D human mesh generation with fine-grained body shape and pose modeling for activity analysis. Inset frames highlight enlarged views of selected body regions for enhanced visualization of pose details.

Although DensePose provides dense spatial correspondences, it does not capture structural dependencies among body parts. Therefore, Mesh Graphormer models the body as a graph and leverages self-attention to learn long-range interactions between anatomically connected regions, enabling robust representation of complex articulated poses. Let (G = (V,E)) denote the body mesh graph, with the graph-attention update defined as:

(26) $$h_{v}^{\left(l+1\right)}=\sigma \left(\sum_{u\in N\left(v\right)}\alpha_{vu}^{\left(l\right)}W^{\left(l\right)}h_{u}^{\left(l\right)}+b^{\left(l\right)}\right)$$

where $N\left(v\right)$ represents the neighborhood of node v. The attention coefficients are computed using scaled dot-product attention:

(27) $$\alpha_{vu}^{\left(l\right)}=\frac{exp\left(\frac{q_{v}^{T}k_{u}}{\sqrt{d_{k}}}\right)}{\sum_{u\prime \in N\left(v\right)}exp\left(\frac{q_{v}^{T}k_{u\prime }}{\sqrt{d_{k}}}\right)}$$

where q and k denote learned query and key projections, respectively. Through iterative message passing and attention aggregation, Mesh Graphormer captures both local body structure and global pose dependencies. The result of the Mesh Graphormer can be depicted in Figure 11.

C. Multi-Model Pose-Flow Fusion (MPFF)

While DensePose and Mesh Graphormer primarily characterize spatial information, activity recognition also requires modeling temporal motion evolution. To capture dynamic behavior, Multi-Model Pose-Flow Fusion (MPFF) is employed. MPFF aggregates pose trajectories obtained from multiple pose estimators, thereby improving robustness against tracking errors, missing joints, and estimator-specific biases. The temporal consistency objective is defined as:

(28) $$S_{MPFF}=\sum_{t}\sum_{j}cos\left(v_{j,t},v_{j,t+1}\right)\cdot \omega_{j,t}$$

where $v_{j,t}$ denotes the velocity of joint j at frame t and $\omega_{j,t}$ represents its confidence score. The final fused trajectory descriptor is computed as:

(29) $$F_{MPFF}=\sum_{m=1}^{M_{p}}\theta_{m}v_{j,t}^{\left(m\right)},\;\;\sum_{m}\theta_{m}=1$$

where $M_{p}$ is the number of pose estimators and $\theta_{m}$ represents reliability weights. Finally, RGB representation is formed by concatenating all features:

(30) $$F_{RGB}=\left[F_{DP};F_{MG};F_{MPFF}\right]$$

**Figure 12:**
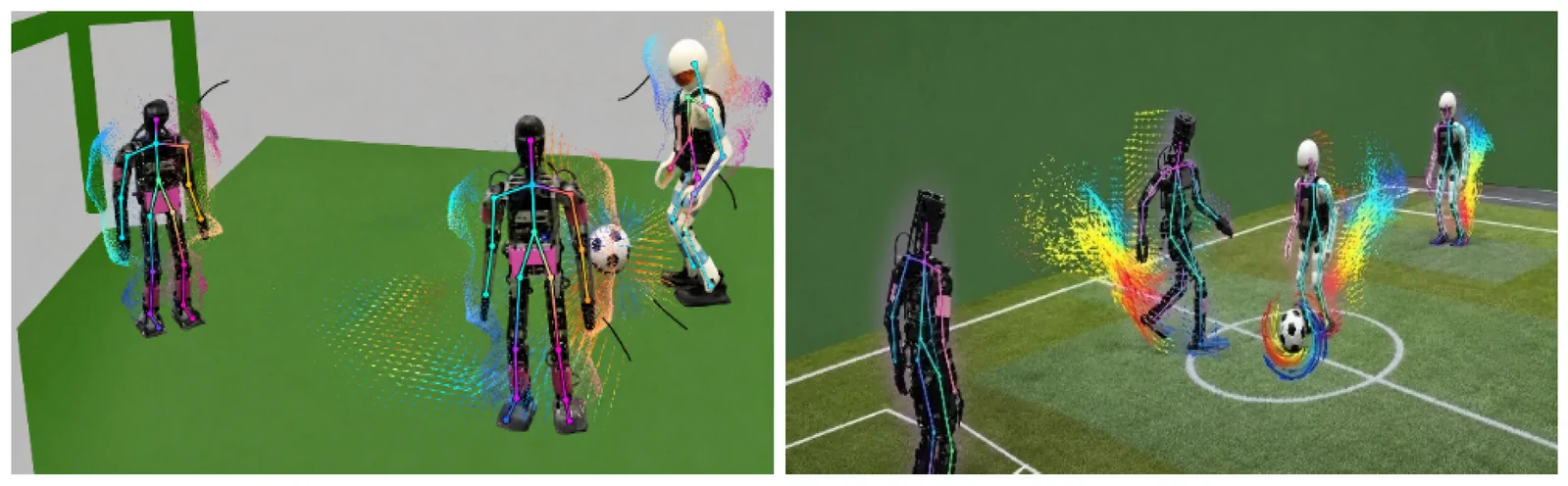
Joint pose-flow feature extraction capturing spatial and temporal dynamics of humanoid robots.

This unified representation integrates spatial structure and temporal dynamics for robust multimodal activity modeling. The result of MPFF can be seen in Figure 12.

### 3.3. Weighted Canonical Feature Fusion and Genetic Optimization

The IMU and RGB [33] branches generate complementary yet heterogeneous representations, with IMU capturing motion dynamics and RGB encoding appearance, pose, dense correspondences, and trajectories. Since direct concatenation can bias learning toward high-dimensional visual features, Weighted Canonical Feature Fusion [34] (WCFF) is employed to learn shared latent structures while balancing modality contributions. Given centered feature matrices FIMU and FRGB, WCFF maximizes the regularized canonical correlation:

(31) $${max}_{u,v}=\frac{u^{T}\Sigma_{IR}v}{\sqrt{\left(u^{T}\left(\Sigma_{II}+\gamma_{I}I\right)u\right)\left(v^{T}\left(\Sigma_{RR}+\gamma_{R}I\right)v\right)}}$$

where $\Sigma_{II}$ and $\Sigma_{RR}$ represent intra-modal covariance matrices, $\Sigma_{IR}$ denotes inter-modal covariance, and $\gamma_{I}$, $\gamma_{R}$ are regularization parameters ensuring numerical stability. The fused representation is constructed as:

(32) $$F_{\mathrm{fused}}=w_{I}\left(U^{T}F_{\mathrm{IMU}}\right)⊕w_{R}\left(V^{T}F_{\mathrm{RGB}}\right),\;w_{I}+w_{R}=1$$

where $w_{I}$ and $w_{R}$ adaptively regulate modality contributions. To further eliminate redundancy and enhance generalization, a Genetic Algorithm (GA) jointly optimizes feature selection and fusion weights. Each chromosome is defined as:

(33) $$g=\left[m_{1},m_{2},\ldots ,m_{D},w_{I},w_{R}\right]$$

The fitness function balances accuracy, sparsity, and diversity:

(34) $$Fit\left(g\right)=A\left(g\right)-\lambda_{g}\frac{∥m{∥}_{0}}{D}+\eta H\left(g\right)$$

where $A\left(g\right)$ denotes validation accuracy, $\lambda_{g}$ controls sparsity, and $H\left(g\right)$ maintains population diversity. Tournament selection, two-point crossover, bit-flip mutation, and elitism are employed for robust optimization. Across both benchmarks, GA converges within 35–40 generations, reducing feature dimensionality by 38–42% and improving validation accuracy by 1.6–2.1%.

**Figure 13:**
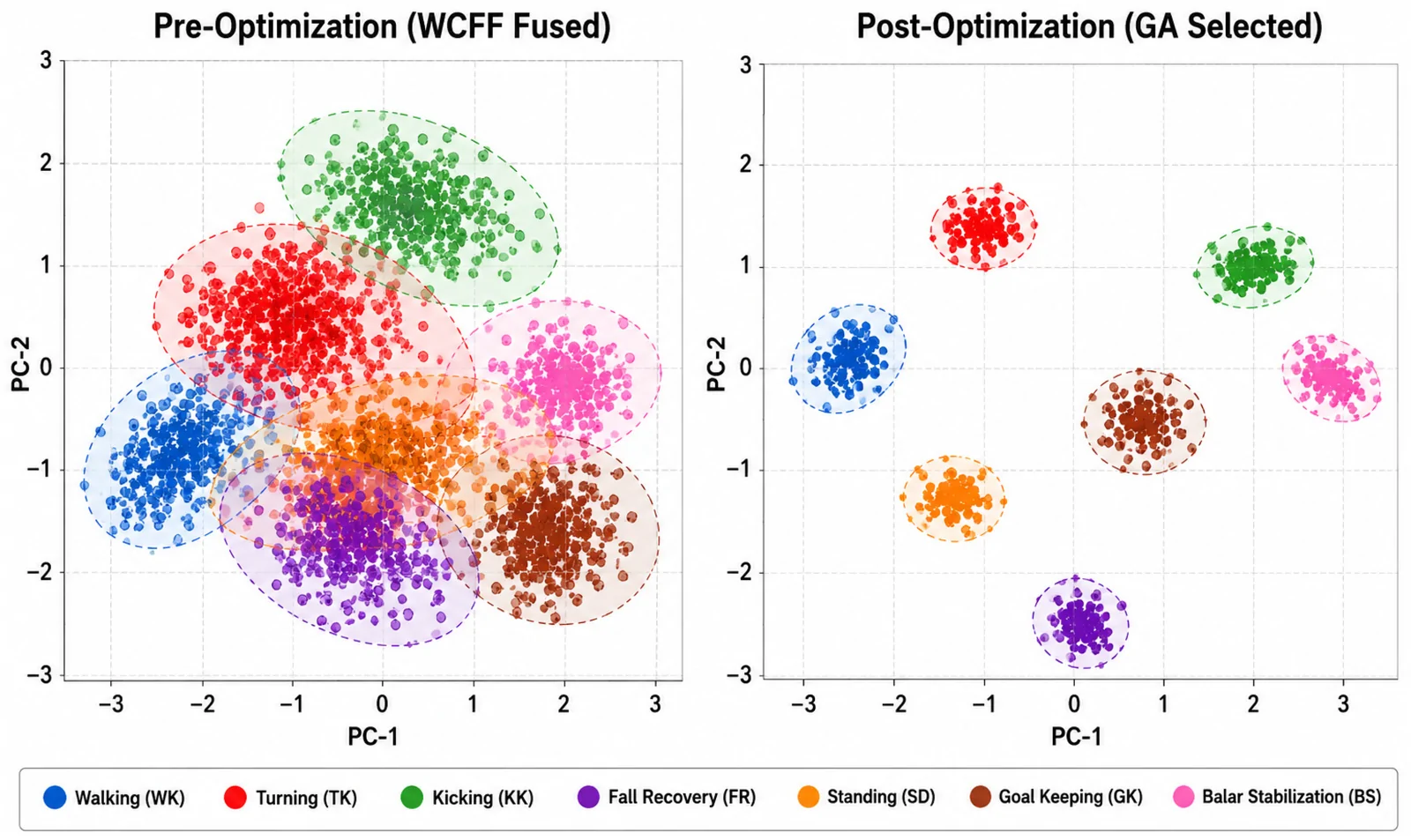
Two-dimensional visualization of the multimodal feature space before and after GA-based feature optimization, showing improved class compactness and inter-class separation following the removal of redundant features.

Figure 13 qualitatively demonstrates the effect of the proposed GA-based feature optimization. Prior to optimization, the WCFF-fused representation exhibits moderate overlap among activities with similar motion patterns, particularly between walking and turning as well as standing and goalkeeping, together with increased intra-class dispersion. Such ambiguity can adversely affect downstream temporal modeling and classification. After GA-based optimization, the selected feature subset yields more compact intra-class clusters and increased inter-class separation by removing redundant and weakly informative features. Although minor boundary ambiguity remains for inherently similar activities, the optimized representation provides a more discriminative feature space for the subsequent Gaussian Process Sequence Model and DeepConvLSTM classifier.

### 3.4. Temporal Alignment via Cluster-Based Sequence Alignment

IMU and RGB streams are inherently asynchronous due to differing sampling rates and processing delays, which leads to temporal misalignment and degrades multimodal fusion. To mitigate this, we introduce Cluster-Based Sequence Alignment (CBSA), which aligns compact motion prototypes instead of raw frames, making it more efficient and robust than Dynamic Time Warping (DTW). First, K-means clustering is applied to obtain motion prototypes $\mu_{k}$ by minimizing intra-cluster variance:

(35) $$J=\sum_{k=1}^{K}\sum_{x_{i}\in C_{k}}∥x_{i}-\mu_{k}{∥}^{2}$$

**Figure 14:**
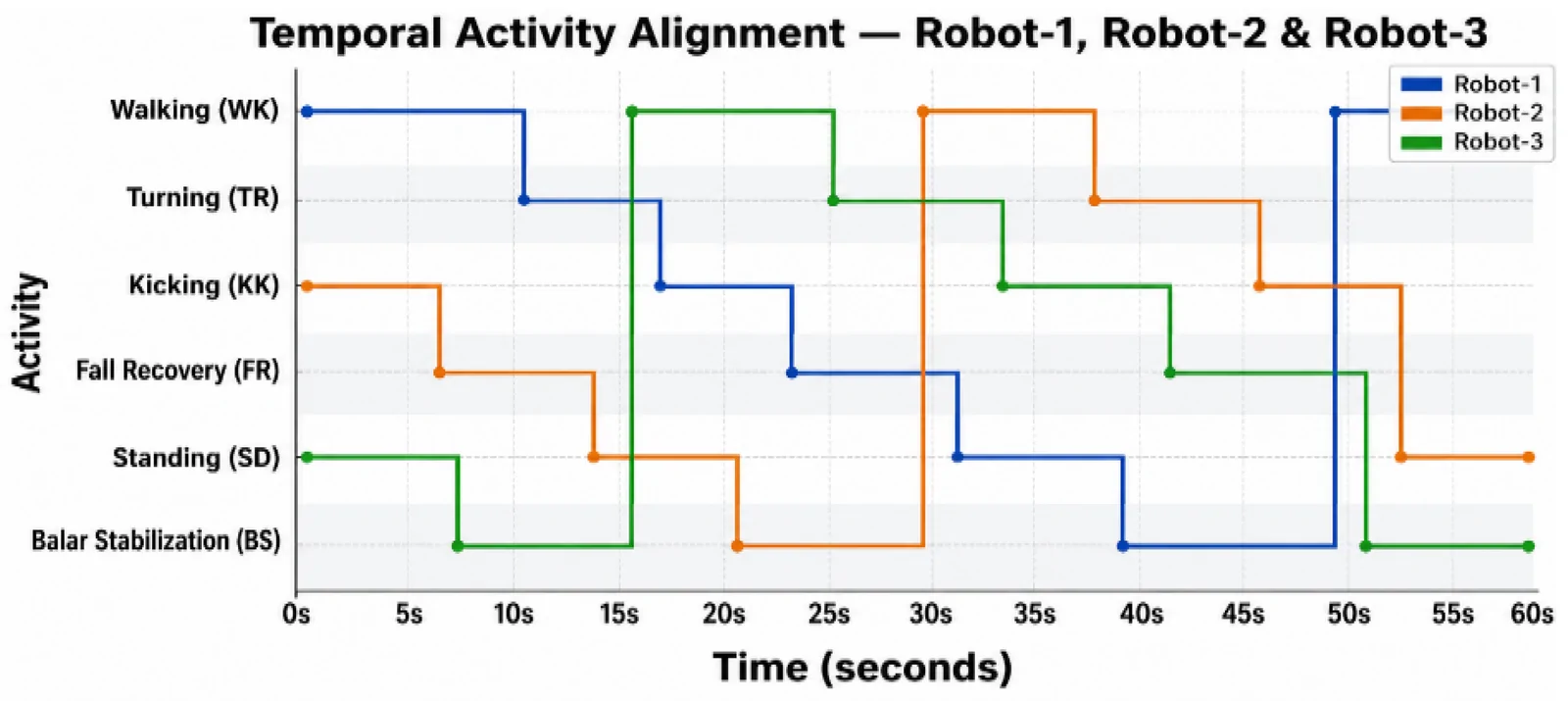
Temporal activity alignment and behavioral state transitions for Robot-1, Robot-2, and Robot-3 over a 60 s duration.

These prototypes replace raw frames, reducing noise and preserving semantically meaningful motion states. Alignment between clustered sequences A and B is then defined as:

(36) $$D\left(A,B\right)={min}_{\pi \in \Pi }\sum_{\left(i,j\right)\in \pi }∥\mu_{a_{i}}-\mu_{b_{j}}{∥}^{2}$$

where Π denotes valid monotonic paths. The optimal alignment is computed via dynamic programming:

(37) $$D\left(i,j\right)=d\left(i,j\right)+min\{D\left(i-1,j\right),D\left(i,j-1\right),D\left(i-1,j-1\right)\}$$

where d(i,j) is the centroid distance. This formulation ensures efficient global alignment in reduced prototype space, producing temporally consistent sequences for downstream activity recognition. The output of the Temporal alignment can be seen in Figure 14.

### 3.5. Activity Mapping via a Gaussian Process Sequence Model

After temporal alignment, the feature trajectories remain highly non-linear and non-stationary due to the complexity of humanoid motion. Deterministic sequence models often fail to capture this variability and provide no uncertainty estimation. To address this, a Gaussian Process Sequence Model (GPSM) is employed for probabilistic temporal modeling. Given time index t and aligned features f, a Gaussian Process prior is assumed:

(38) $$f∼GP\left(\mu \left(t\right),k\left(t,t\prime \right)\right)$$

using a squared-exponential kernel:

(39) $$k\left(t,t\prime \right)=\sigma_{f}^{2}exp\left(-\frac{{\left(t-t\prime \right)}^{2}}{2l^{2}}\right)$$

This kernel ensures smooth temporal modeling while suppressing noise. The predictive mean and variance are given by:

(40) $$\mu^{*}\left(t^{*}\right)=k_{*}^{T}{\left(K+\sigma_{n}^{2}I\right)}^{-1}f,\;\sigma_{*}^{2}\left(t^{*}\right)=k\left(t^{*},t^{*}\right)-k_{*}^{T}{\left(K+\sigma_{n}^{2}I\right)}^{-1}k_{*}$$

**Figure 15:**
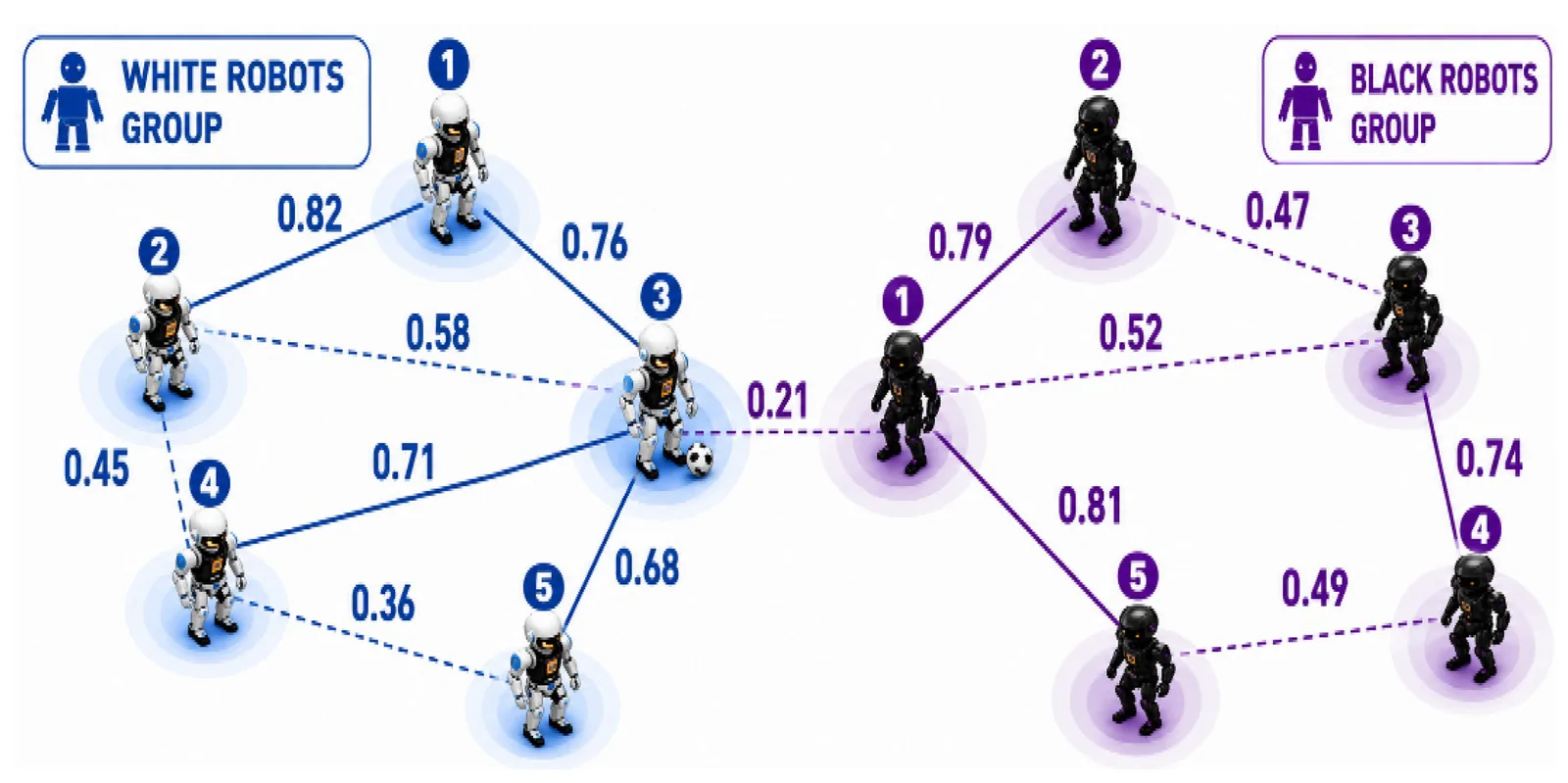
Interaction graph between the White Robots Group and Black Robots Group. Blue and purple colors denote the two robot groups, respectively; solid and dashed lines represent strong and weak interaction links, while the numbers on the edges indicate the corresponding interaction weights.

The model is trained by maximizing the marginal log-likelihood, enabling a balance between data fit and temporal smoothness. This yields a refined latent representation $F_{GP}$, which is robust to noise and temporal inconsistencies. Finally, $F_{GP}$ is passed to the DeepConvLSTM classifier for activity recognition. Figure 15 illustrates the interaction graph between the White Robots Group and Black Robots Group, where weighted connections represent the interaction strength among robots within each group.

### 3.6. Classification via DeepConvLSTM

The final stage employs DeepConvLSTM for activity classification, combining convolutional feature extraction with recurrent temporal modeling to capture both local motion patterns and long-term dependencies. The convolutional layers learn localized temporal features as:

(41) $$c_{l}^{\left(j\right)}=\sigma \left(\sum_{i}W_{l}^{\left(ij\right)}*c_{l-1}^{\left(i\right)}+b_{l}^{\left(j\right)}\right)$$

The extracted features are then processed by stacked LSTM layers to model temporal dynamics:

(42) $$i_{t}=\sigma \left(W_{i}x_{t}+U_{i}h_{t-1}\right),\;f_{t}=\sigma \left(W_{f}x_{t}+U_{f}h_{t-1}\right),\;o_{t}=\sigma \left(W_{o}x_{t}+U_{o}h_{t-1}\right)c_{t}=f_{t}⊙c_{t-1}+i_{t}⊙tanh\left(W_{c}x_{t}+U_{c}h_{t-1}\right),\;h_{t}=o_{t}⊙tanh\left(c_{t}\right)$$

The final hidden state $h_{T}$ is converted into class probabilities using the following:

(43) $$p\left(y|F_{GP}\right)=softmax\left(W_{y}h_{T}+b_{y}\right)$$

The model is trained using categorical cross-entropy with L2 regularization:

(44) $$L=-\sum_{c=1}^{C}y_{c}logp_{c}+\zeta \sum_{l}∥\Theta_{l}{∥}_{2}^{2}$$

**Figure 16:**
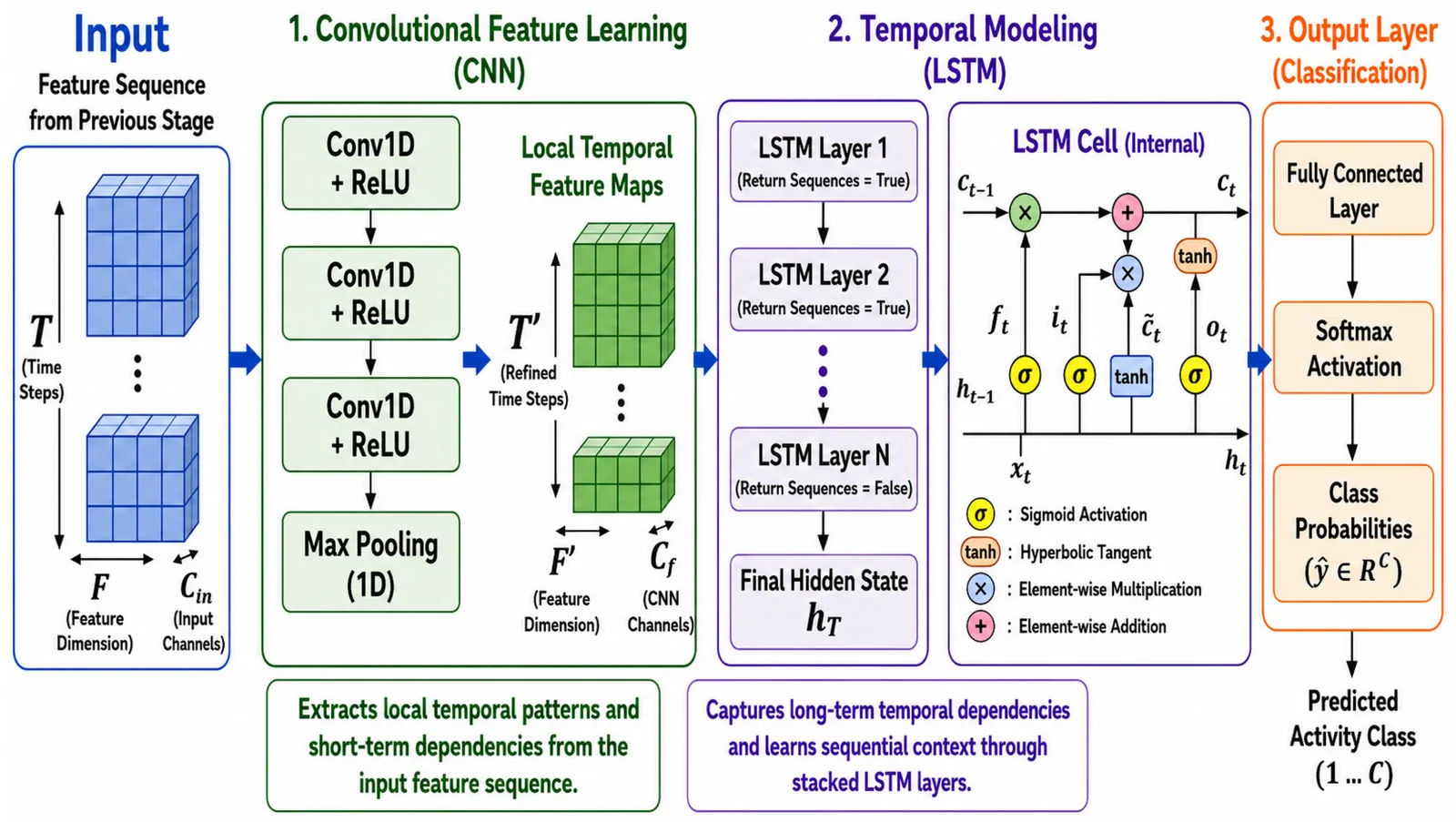
Proposed CNN–LSTM architecture for temporal feature learning and activity classification. Arrows indicate the data flow between processing stages, while colors distinguish the input, CNN feature extraction, LSTM temporal modeling, and output classification modules.

This architecture jointly learns spatial-temporal representations, enabling robust and accurate multimodal humanoid activity recognition. Figure 16 presents the proposed CNN–LSTM framework, in which local temporal features extracted by the CNN are sequentially modeled by stacked LSTM layers before final activity classification.

## 4. Experimental Section

### 4.1. System Specifications

All experiments were conducted on a workstation equipped with an Intel® Core™ i9-13900 K (Intel Corporation, Santa Clara, CA, USA) CPU running at 3.0 GHz with 24 cores, 64 GB DDR5 memory, and an NVIDIA RTX 4090 GPU (NVIDIA Corporation, Santa Clara, CA, USA) with 24 GB VRAM under Ubuntu 22.04 LTS (Canonical Ltd., London, UK). The framework was implemented in Python 3.10 (Python Software Foundation, Wilmington, DE, USA) using PyTorch 2.2.0 (Linux Foundation AI & Data, San Francisco, CA, USA) with CUDA 12.1 (NVIDIA Corporation, Santa Clara, CA, USA), scikit-learn 1.4 (Inria, Paris, France) (KELM, KCCF, GA), sktime 0.27 (Alan Turing Institute, London, UK) (MINIROCKET, TS-CHIEF, r-STSF), MMPose 1.2.0 (OpenMMLab, Shanghai, China) (HRNet, DensePose R-CNN, Mesh Graphormer), and OpenCV 4.9 (OpenCV.org, Palo Alto, CA, USA). Random seeds were fixed at 42 for reproducibility, and all reported results represent the mean of three independent runs.

### 4.2. Dataset Description

Two humanoid movement benchmarks were employed as a controlled, reproducible, and privacy-preserving validation platform for assessing the proposed sensor-fusion framework prior to human-subject deployment. Both datasets provide visual streams accompanied by reliable ground-truth annotations; however, neither includes complete inertial recordings suitable for the inertial processing branch. SoccerDiffusion retains only reconstructed roll and pitch due to a recording fault, whereas HumanoidRobotPose does not contain inertial measurements. Accordingly, the inertial modality considered in this study was established under the adopted experimental protocol to enable the evaluation of multimodal fusion, evolutionary optimization, and temporal modeling within a consistent experimental setting. The resulting performance therefore reflects the behavior of the proposed framework under the adopted evaluation protocol rather than deployment with physical inertial sensors.

#### 4.2.1. Soccer Diffusion Dataset

The SoccerDiffusion dataset is a large-scale humanoid robot soccer benchmark derived from RoboCup KidSize League recordings. It comprises 88 matches spanning approximately 15.1 h of gameplay, containing over 523,000 RGB frames and 2.65 million synchronized motion samples. The dataset provides ego-centric RGB video with a resolution of 480 × 480 pixels together with robot joint and kinematic information. It captures a diverse range of soccer-specific humanoid activities, including walking, turning, kicking, goalkeeping, ball stabilization, and fall recovery, performed in realistic multi-agent environments. These characteristics make SoccerDiffusion a valuable benchmark for evaluating humanoid activity recognition and multimodal perception frameworks.

#### 4.2.2. HumanoidRobotPose Dataset

The HumanoidRobotPose dataset is an RGB-based benchmark developed for humanoid robotic pose estimation and activity recognition. It contains annotated humanoid robot activities captured from multiple viewpoints under diverse environmental conditions, enabling robust evaluation of vision-based perception algorithms. The dataset comprises approximately 1.5 K annotated RGB images in COCO keypoint format and includes ten activity classes: walking, standing, sitting, turning, arm movement, front pose, side pose, back pose, dynamic motion, and partial occlusion pose. Its diverse poses, viewpoints, and activity variations make it a valuable benchmark for evaluating humanoid pose estimation and activity recognition frameworks.

### 4.3. Evaluation Protocol and Performance Analysis

An 80/20 subject-independent train–test split was employed to prevent data leakage. Performance was evaluated using accuracy, precision, recall, F1-score, ROC–AUC, confusion matrices, and inter-fold standard deviation. The confusion matrices (Table 1 and Table 2) exhibit strong diagonal dominance, with most errors occurring between visually similar motion classes, while distinct activities achieve higher recognition accuracy. Per-class precision, recall, and F1-score (Table 3 and Table 4) further demonstrate consistent and balanced performance across all classes, with only slight reductions for similar manipulation primitives due to moderate class imbalance.

### 4.4. ROC and AUC Analysis

**Figure 17:**
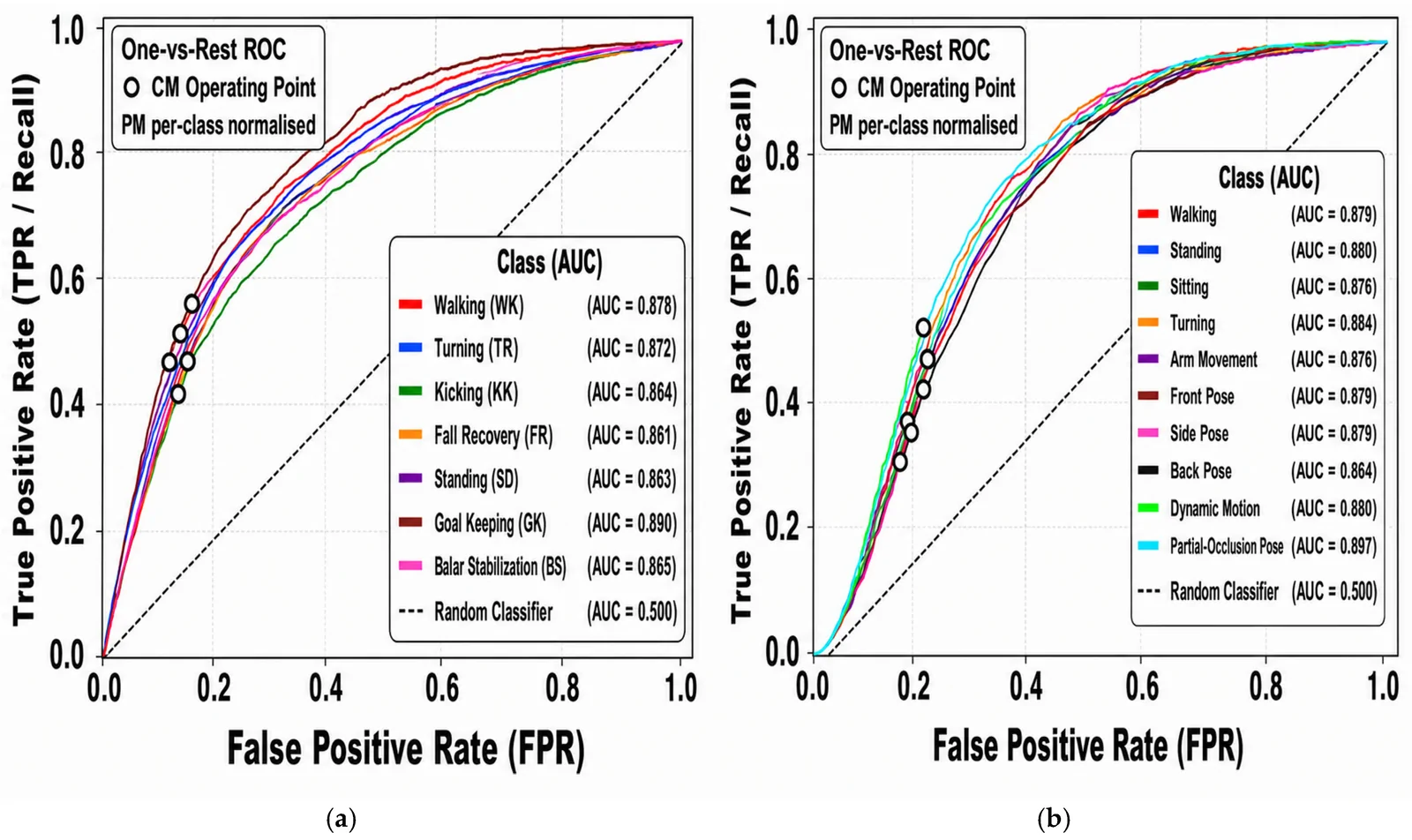
(**a**) Per-class ROC curves on the SoccerDiffusion (one-vs-rest); (**b**) HumanoidRobotPose dataset.

Receiver Operating Characteristic (ROC) curves were generated in a one-vs-rest fashion for each activity class. Figure 17 presents the ROC curves on the SoccerDiffusion and HumanoidRobotPose datasets.

### 4.5. Robustness Evaluation via Five-Fold Cross-Validation and Multi-Seed Analysis

To further evaluate the robustness and reproducibility of the proposed multimodal framework beyond a single train−test split, additional experiments were conducted using five-fold subject-independent cross-validation and multi-seed analysis. In the cross-validation protocol, the dataset was partitioned into five mutually exclusive folds while ensuring subject independence to prevent data leakage. During each iteration, four folds were used for training, and the remaining fold was reserved for testing. Performance was then averaged across all folds, and the corresponding standard deviation was computed to quantify model stability.

Furthermore, to assess the influence of stochastic optimization and weight initialization, the proposed framework was independently trained using five different random seeds (42, 7, 123, 2024, and 31,337). All architectural configurations, optimization settings, and hyperparameters were kept unchanged across experiments so that only the randomness associated with initialization and data shuffling affected the training process.

The five-fold cross-validation results presented in Table 5 demonstrate that the proposed framework consistently achieves high recognition performance across different data partitions. On the SoccerDiffusion dataset, the framework attained an average accuracy of 86.56 ± 0.44% with a Macro-F1 score of 86.19 ± 0.42%, whereas on the HumanoidRobotPose dataset, it achieved an average accuracy of 88.04 ± 0.46% and a Macro-F1 score of 87.75 ± 0.47%. Although slight variations are observed among individual folds, these fluctuations remain relatively small and primarily reflect differences in activity complexity and subject variability across the evaluation partitions. The consistently low standard deviations indicate that the proposed multimodal fusion strategy generalizes well across different subsets of the data rather than relying on a favorable train−test split.

Similarly, the multi-seed evaluation results summarized in Table 6 further confirm the robustness of the proposed framework. Despite using different random seeds during network initialization and optimization, the obtained accuracies remained highly consistent, yielding average accuracies of 86.56 ± 0.31% on SoccerDiffusion and 88.03 ± 0.25% on HumanoidRobotPose. The limited variation across independent training runs demonstrates that the proposed framework is relatively insensitive to stochastic initialization, indicating stable optimization behavior and reliable convergence. These findings collectively verify that the reported performance is reproducible and is not the consequence of a favorable random initialization or a particular data partition.

### 4.6. Leakage-Free Experimental Pipeline

To ensure an unbiased evaluation, a strict subject-independent experimental protocol was adopted throughout the entire pipeline. The dataset was partitioned into training and testing subsets before any learnable preprocessing, feature optimization, or model training was performed, ensuring that no subject appeared in both partitions. Consequently, the testing data remained completely unseen during model development.

All preprocessing stages involving parameter estimation were fitted exclusively using the training data. In particular, the KELM denoising model, KCCF kernel transformations, WCFF fusion parameters, Genetic Algorithm feature selection, Gaussian Process Sequence Model, and DeepConvLSTM classifier were learned only from the training partition. The transformations learned were subsequently applied to the corresponding testing samples without further optimization or parameter updates.

Operations that do not require dataset-level statistics, including entropy-monitoring windowing and sequence segmentation, were performed independently on each individual sample and therefore did not introduce information leakage between the training and testing partitions. Likewise, no normalization statistics, optimization parameters, or feature-selection decisions were computed using the complete dataset. This protocol guarantees that the validation and testing samples remained entirely independent throughout all stages of preprocessing, feature extraction, multimodal fusion, optimization, and classification. Table 7 summarizes the leakage prevention strategy adopted in the proposed pipeline. 

### 4.7. Baseline Comparison and Fusion Ablation

To verify that the observed performance improvements originate from the proposed multimodal fusion strategy rather than increased classifier capacity, additional baseline comparisons and fusion ablation experiments were conducted.

First, Table 8 extends the comparison by incorporating two state-of-the-art vision-only Transformer models, TimeSformer [20] and VideoMAE V2 [32], which operate solely on raw RGB video sequences without inertial information. These models complement the IMU-only baselines, DeepConvLSTM [35] and IMU-Transformer [36], thereby providing representative unimodal baselines for both visual and inertial modalities. To ensure a fair evaluation, all baselines were assessed using the identical 5-fold subject-independent partitions, random seed, training protocol, hyperparameter optimization strategy, and fine-tuning procedure adopted for the proposed framework. The vision-only models were trained directly on raw RGB frames, allowing them to serve as independent baselines rather than variants of the proposed RGB processing branch. The fusion ablation analysis with a fixed classifier is summarized in Table 9.

### 4.8. Computational Complexity

Table 10 summarizes the asymptotic computational complexity (Big-O) and measured wall-clock time for each stage of the proposed pipeline on a single input segment of length T = 200 with feature dimension D = 256. The dominant costs come from KCCF and DeepConvLSTM, but both remain well within real-time budgets on the workstation hardware described in Section 4.1.

## 5. Discussion

The experimental results demonstrate that hierarchically fusing wearable inertial and visual sensing, rather than relying on either modality alone, yields consistent and substantial gains in movement-recognition accuracy. On the two humanoid movement benchmarks, the proposed framework attained an accuracy of 86.56% on SoccerDiffusion and an accuracy of 88.04% on HumanoidRobotPose, exceeding the strongest multimodal baseline (RoboHAR) by 1.59 and 1.31 percentage points, respectively, and the inertial-only DeepConvLSTM baseline by 7.13 and 6.82 percentage points, respectively (Table 5). The magnitude of the improvement over unimodal pipelines confirms that inertial and visual cues are genuinely complementary: the IMU branch captures fine-grained temporal dynamics that are difficult to recover from video under motion blur, while the visual branch supplies pose, surface, and trajectory information that disambiguates motions which are kinematically similar at the sensor level. This complementarity is precisely what movement-health applications demand, where superficially similar gait or limb patterns can correspond to clinically distinct compensation strategies.

Component-wise analysis indicates that this performance is not attributable to any single module but to the joint contribution of representation learning, evolutionary optimization, and temporal modeling. Genetic Algorithm-based feature selection converged within 35–40 generations while reducing feature dimensionality by 38–42% and improving validation accuracy by 1.6–2.1%, showing that much of the fused representation is redundant and that discriminative compaction both lightens the model and sharpens class separability. The confusion matrices (Table 1 and Table 2) exhibit strong diagonal dominance, with residual errors concentrated among visually similar movement classes—an error structure that is comparatively benign for triage-oriented telerehabilitation, where the clinically salient distinction is typically between well-executed and aberrant movement rather than between two closely related correctly executed actions.

From a translational standpoint, the architecture maps naturally onto sensor-driven telerehabilitation and athlete-monitoring workflows. The wearable IMU branch corresponds to body-worn sensors that an athlete or patient can use unsupervised at home; the markerless RGB branch corresponds to a single consumer-grade camera, avoiding marker-based motion capture; and the cluster-based temporal alignment directly addresses the asynchrony that arises when wearable and camera streams are captured on independent devices with differing sampling rates. The Gaussian Process Sequence Model additionally yields calibrated predictive uncertainty, which is valuable in a remote setting where a system should defer ambiguous cases to a clinician rather than issue an overconfident automated assessment. Embedded within a clinical workflow, the resulting quantitative movement-data analysis could inform more reliable clinical decisions, enable objective tracking of recovery trajectories and support data-driven adjustment of individualized treatment plans. Together, these properties align the framework with the broader goal of AI-driven sensing technologies for remote, continuous, and patient-centric monitoring.

Several limitations should be acknowledged. Most importantly, validation was conducted on humanoid movement benchmarks rather than on human participants; while humanoid platforms provide a controlled, repeatable, and privacy-preserving testbed with reliable ground truth, the kinematic and morphological differences between humanoid and human movement mean that the reported accuracies should be interpreted as evidence of methodological soundness rather than as clinical performance. Prospective evaluation on instrumented human cohorts—spanning healthy athletes, injury-prevention screening, and post-operative recovery populations—is required before any clinical or sports-medicine deployment. Additional limitations include the cubic-time cost of the KELM and Gaussian Process stages, which, although within real-time budgets on the workstation hardware used here, would benefit from low-rank or sparse approximation for embedded wearable deployment.

Future work will therefore prioritize human-subject data collection and clinical validation, lightweight on-device inference for resource-constrained wearables, and end-to-end differentiable optimization to replace the staged feature-selection pipeline. Incorporating complementary modalities such as surface electromyography and depth sensing would further enrich the neuromotor characterization available to remote-assessment systems.

### Limitations

Although the proposed multimodal framework demonstrates promising performance, several limitations should be considered when interpreting the reported results. The experimental evaluation is conducted on benchmark datasets that do not provide complete inertial recordings, requiring the inertial modality to be established according to the adopted experimental protocol. Consequently, the reported fusion results reflect the behavior of the proposed framework within the adopted evaluation setting. Further validation using datasets containing synchronized visual and real inertial measurements would provide additional evidence of the framework’s applicability under practical sensing conditions.

First, although the proposed preprocessing pipeline incorporates Kernel Extreme Learning Machine (KELM) denoising and anisotropic diffusion filtering to improve signal quality, the present study does not include a dedicated robustness evaluation under controlled sensor degradation. The benchmark datasets were collected from real-world humanoid robotic platforms operating under structured experimental conditions and therefore do not comprehensively represent challenging deployment scenarios involving severe sensor noise, signal dropout, mounting variations, or visual degradation. Consequently, the robustness of the proposed framework under such adverse conditions remains an important direction for future investigation through controlled noise-injection and perturbation studies.

Second, the computational performance of the proposed framework has been evaluated using a high-performance workstation environment, which is appropriate for algorithmic validation but does not directly reflect deployment on embedded or resource-constrained robotic platforms. Although the proposed pipeline demonstrates practical computational efficiency for offline and workstation-based applications, additional optimization strategies, including lightweight feature extraction, model compression, pruning, knowledge distillation, and efficient multimodal fusion, should be investigated before real-time edge deployment can be fully established. Accordingly, no claim regarding embedded real-time implementation is made in this study.

Third, while the proposed framework was validated on two publicly available benchmark datasets containing real-world humanoid robot recordings, its robustness under substantial variations in camera viewpoints, sensor placement, robot morphology, and hardware configurations has not been systematically evaluated. Although the adopted multimodal architecture improves representation learning by integrating complementary inertial and visual information, explicit viewpoint-invariant learning and sensor-placement adaptation were beyond the scope of the present work. Future studies will therefore investigate cross-view evaluation protocols, sensor-placement perturbation analysis, and domain adaptation strategies to further improve the generalization capability of the proposed framework across diverse robotic platforms and real-world rehabilitation environments.

## 6. Conclusions

This paper presented a hierarchical multimodal framework for sensor-driven movement analysis, integrating wearable IMU signals and RGB visual information through kernelized representation learning, ensemble time-series feature extraction, dense visual feature extraction, Weighted Canonical Feature Fusion, Genetic Algorithm optimization, cluster-based temporal alignment, Gaussian Process activity mapping, and DeepConvLSTM classification. Under an 80/20 subject-independent train–test split, the framework achieved an accuracy of 86.56% on the SoccerDiffusion benchmark and an accuracy of 88.04% on the HumanoidRobotPose benchmark, consistently outperforming inertial-only, vision-only, and naïvely fused state-of-the-art baselines, while operating within real-time budgets on commodity GPU hardware. Because these benchmarks comprise humanoid platforms rather than human participants, the results establish the methodological soundness of the fusion-and-optimization pipeline and should be read as a controlled, privacy-preserving precursor to clinical evaluation rather than as a demonstration of clinical accuracy. The architecture, nonetheless, maps directly onto the sensing requirements of sports telerehabilitation and athlete monitoring—body-worn inertial units, a single markerless camera, robustness to cross-device asynchrony, and uncertainty-aware prediction—positioning it as a foundation for AI-assisted, home-based movement assessment. Future research will prioritize human-subject validation across injury-prevention and post-operative recovery cohorts, lightweight on-device inference for resource-constrained wearables, end-to-end differentiable feature optimization, and the incorporation of complementary modalities such as surface electromyography and depth sensing to enrich neuromotor characterization.

## Figures and Tables

**Table 1:** Confusion matrix for the SoccerDiffusion dataset (per-class recognition rates, %).

Actual/Predicted	WK	TR	KK	FR	SD	GK	BS
Walking (WK)	85	4	5	2	1	2	1
Turning (TR)	5	84	3	3	2	2	1
Kicking (KK)	5	4	83	3	2	2	1
Fall Recovery (FR)	2	3	3	86	3	2	1
Standing (SD)	2	2	3	3	87	2	1
Goal Keeping (GK)	2	2	1	2	2	88	3
Balar Stabilization (BS)	1	1	1	1	1	2	93

**Table 2:** Confusion matrix for the HumanoidRobotPose dataset (per-class recognition rates, %).

Actual/Predicted	Wlk	Std	Sit	Trn	ArM	FrP	SdP	BkP	DyM	POP
Walking (Wlk)	87	2	1	3	2	1	1	1	1	1
Standing (Std)	2	86	3	1	2	2	1	1	1	1
Sitting (Sit)	1	3	87	1	2	2	1	1	1	1
Turning (Trn)	3	1	1	88	2	1	1	1	1	1
Arm Movement (ArM)	1	2	1	1	89	2	1	1	1	1
Front Pose (FrP)	1	2	1	1	2	87	2	2	1	1
Side Pose (SdP)	1	1	1	1	2	2	88	2	1	1
Back Pose (BkP)	1	1	1	1	2	2	2	86	2	2
Dynamic Motion (DyM)	1	1	1	1	2	1	1	2	89	1
Partial-Occlusion (POP)	1	1	1	1	1	1	1	1	1	91

**Table 3:** Precision, recall, and F1-score on the SoccerDiffusion dataset.

Class	Precision (%)	Recall (%)	F1-Score (%)
Walking (WK)	83.33	85.00	84.16
Turning (TR)	84.00	84.00	84.00
Kicking (KK)	83.84	83.00	83.42
Fall Recovery (FR)	86.00	86.00	86.00
Standing (SD)	88.78	87.00	87.88
Goal Keeping (GK)	88.00	88.00	88.00
Balar Stabilization (BS)	92.08	93.00	92.54
Macro Average	86.58	86.57	86.57

**Table 4:** Precision, recall, and F1-score on the HumanoidRobotPose dataset.

Class	Precision (%)	Recall (%)	F1-Score (%)
Walking	87.00	87.00	87.00
Standing	86.00	86.00	86.00
Sitting	87.87	87.00	87.43
Turning	88.88	88.00	88.43
Arm Movement	85.57	89.00	87.25
Front Pose	86.13	87.00	86.56
Side Pose	88.00	88.00	88.00
Back Pose	87.75	86.00	86.86
Dynamic Motion	89.89	89.00	89.44
Partial-Occlusion Pose	91.00	91.00	91.00
Macro Average	87.81	87.80	87.80

**Table 5:** Five-fold subject-independent cross-validation results of the proposed multimodal framework on the SoccerDiffusion and HumanoidRobotPose datasets (mean ± standard deviation).

Fold	SoccerDiffusion Acc.	SoccerDiffusion Macro-F1	HumanoidRobotPose Acc.	HumanoidRobotPose Macro-F1
Fold 1	85.92	85.55	87.43	87.12
Fold 2	86.48	86.10	88.11	87.81
Fold 3	87.08	86.73	88.63	88.36
Fold 4	86.77	86.38	88.29	88.01
Fold 5	86.55	86.18	87.74	87.46
Mean ± SD	86.56 ± 0.44	86.19 ± 0.42	88.04 ± 0.46	87.75 ± 0.47

**Table 6:** Robustness analysis of the proposed framework under different random seed initializations, demonstrating the stability and reproducibility of the reported recognition performance.

Seed	SoccerDiffusion	HumanoidRobotPose
42	86.56	88.04
7	86.14	87.69
123	86.83	88.31
2024	86.37	87.91
31,337	86.91	88.22
Mean ± SD	86.56 ± 0.31	88.03 ± 0.25

**Table 7:** Leakage prevention strategy adopted throughout the experimental pipeline.

Pipeline Stage	Training Data Only	Test Data Used During Fitting	Leakage Risk	Mitigation Strategy
Subject-independent split	✓	No	None	Dataset partitioned before any learnable operation.
KELM preprocessing	✓	No	Low	KELM parameters estimated exclusively from the training partition and applied unchanged to the test set.
KCCF kernel learning	✓	No	Low	Kernel mappings are learned only from training samples.
Entropy-monitoring windowing	N/A	No	None	Applied independently to each signal without dataset-level statistics.
MINIROCKET/TS-CHIEF/r-STSF	✓	No	Low	Feature extraction fitted only on training data.
WCFF fusion	✓	No	Low	Fusion weights are estimated exclusively using training features.
Genetic Algorithm	✓	No	Medium	Feature selection optimized only on the training partition.
GPSM	✓	No	Low	Model trained exclusively on training sequences.
DeepConvLSTM	✓	No	None	Network optimized only using the training partition.

Where ✓ indicates that the corresponding feature or component is included in the method.

**Table 8:** Performance comparison with representative unimodal and multimodal state-of-the-art methods.

Method	Modality	Soccer Diffusion Acc. (%)	HumanoidRobotPose Acc. (%)
DeepConvLSTM [35]	IMU	79.43	81.22
Transformer-based diffusion model [36]	IMU	81.05	82.87
TimeSformer [20]	RGB (Vision Transformer)	82.18	83.76
VideoMAE V2 [32]	RGB (Masked Video Transformer)	83.54	85.08
HAMLET [37]	IMU + RGB	82.60	83.94
BodyFormer [38]	IMU + Skeleton	83.78	85.31
HARFusion [39]	IMU + RGB	84.12	86.09
HRNet [40]	IMU + Video	84.97	86.73
Proposed Method	IMU + RGB	86.56	88.04

**Table 9:** Fusion ablation with the classifier held constant.

Configuration	Classifier	Soccer Diffusion Acc. (%)	HumanoidRobotPose Acc. (%)
(1) IMU branch only	DeepConvLSTM	81.20	83.05
(2) RGB branch only	DeepConvLSTM	82.67	84.10
(3) Naïve concatenation	DeepConvLSTM	83.75	85.20
(4) WCFF fusion, without GA	DeepConvLSTM	84.89	86.15
(5) WCFF + GA (proposed)	DeepConvLSTM	86.56	88.04

**Table 10:** Computational complexity (Big-O) and per-segment wall-clock time of each pipeline stage.

Stage	Time Complexity	Space Complexity	Per-Segment Time (ms)
KELM Preprocessing	O(T^3^)	O(T^2^)	8.4
KCCF Multikernel Fusion	O(M T^3^)	O(M T^2^)	21.3
Entropy-Monitoring Windowing	O(T B)	O(B)	1.2
MINIROCKET	O(N T)	O(N)	3.6
TS-CHIEF	O(K_t_ T log T)	O(K_t_ T)	12.5
r-STSF	O(K_r_ T log T)	O(K_r_ T)	9.8
Anisotropic Diffusion	O(n H W)	O(H W)	5.7
HRNet Silhouette	O(H W C)	O(H W C)	18.2
DensePose R-CNN	O(H W C)	O(H W C)	26.8
Mesh Graphormer	O(|V|^2^ d)	O(|V| d)	22.4
MPFF	O(J T)	O(J T)	4.1
WCFF	O(D^3^)	O(D^2^)	11.5
Genetic Algorithm	O(G P D)	O(P D)	14.7
Cluster-based Sequence Alignment	O(K T)	O(K T)	6.2
Gaussian Process Sequence Model	O(T^3^)	O(T^2^)	9.1
DeepConvLSTM	O(T D h + T h^2^)	O(T h)	23.6

## Data Availability

The two benchmark datasets used for evaluation in this study are publicly available. The SoccerDiffusion dataset can be accessed at https://bit-bots.github.io/SoccerDiffusion (Accessed on 7 April 2026). The HumanoidRobotPose dataset is publicly available at https://github.com/AIS-Bonn/HumanoidRobotPoseEstimation. (Accessed on 25 May 2026).
